# YBX1 Expression Marks Proliferative Tumour States with Context-Dependent Genomic Instability: A Pan-Cancer Analysis

**DOI:** 10.3390/ijms27104340

**Published:** 2026-05-13

**Authors:** Selena Wang, Zahra Shafaei Pishabad, Debina Sarkar, Apeksha Arun Bhandarkar, Makhdoom Sarwar, Aaron Jeffs, Glen Reid, Antony Braithwaite, Sunali Mehta

**Affiliations:** 1 Department of Pathology and Molecular Medicine, University of Otago, Dunedin 9016, New Zealand; wanse679@student.otago.ac.nz (S.W.); zahra.shafaei@otago.ac.nz (Z.S.P.); debina.sarkar@otago.ac.nz (D.S.); bhaap437@student.otago.ac.nz (A.A.B.); aaron.jeffs@otago.ac.nz (A.J.); glen.reid@otago.ac.nz (G.R.); antony.braithwaite@otago.ac.nz (A.B.); 2 Maurice Wilkins Centre for Biodiscovery, University of Auckland, Auckland 1142, New Zealand; mak.sarwar@otago.ac.nz; 3 Department of Obstetrics and Gynaecology, University of Otago, Christchurch 8011, New Zealand; 4 School of Pharmacy and Biomedical Sciences, The University of Waikato Te Whare Wananga o Waikato, Hamilton 3240, New Zealand

**Keywords:** YBX1, YB-1, pan-cancer, genomic instability, chromosomal instability

## Abstract

Y-box binding protein 1 (YB-1; encoded by *YBX1*) is a multifunctional DNA- and RNA-binding protein implicated in cell cycle regulation, DNA repair, stress adaptation, and therapy resistance. Elevated *YBX1* expression has been associated with aggressive disease across multiple cancer types; however, its pan-cancer genomic and clinical correlates, and the extent to which these reflect proliferative activity versus genomic instability, remain incompletely defined. Here, we performed an integrative pan-cancer analysis across 53 independent datasets spanning 33 tumour types, incorporating transcriptomic (*YBX1* mRNA), proteomic (RPPA), genomic, and clinical data. We found that *YBX1* is rarely altered at the genomic level, whereas its mRNA expression is highly variable within tumour cohorts. Tumours with high *YBX1* mRNA expression consistently exhibited conserved transcriptional programmes enriched for cell cycle, mitotic, RNA processing, and signalling pathways, patterns that were also reflected at the protein level by concordant pathway associations with elevated YB-1 abundance. These molecular features co-occurred with clinicopathological characteristics indicative of aggressive disease. High *YBX1* mRNA expression was associated with increased mutation burden, chromosomal alteration burden, hypoxia, and homologous recombination deficiency at the pan-cancer level, with similar molecular associations observed in tumours stratified by elevated YB-1 protein levels. The association between *YBX1* expression and chromosomal alteration burden was largely attenuated after accounting for proliferative activity, particularly G2/M-associated transcriptional programmes used as a proxy for mitotic activity. While the relationship with mutation burden was heterogeneous across tumour types, this pattern suggests that links between *YBX1* expression and chromosomal instability primarily reflect shared proliferative and mitotic tumour biology rather than an independent genomic instability programme. Clinically, high *YBX1* mRNA expression was associated with advanced disease stage, higher histologic grade, reduced progression-free survival, and poorer overall survival. Elevated YB-1 protein levels were also associated with advanced disease stage and poorer survival outcomes and demonstrated a similar, although non-significant, directional trend with histologic grade. Collectively, these findings demonstrate that elevated *YBX1* expression marks proliferative and clinically aggressive tumour states within which genomic instability-related features arise in a context-dependent manner, providing a clarified pan-cancer framework for interpreting YB-1-associated tumour biology.

## 1. Introduction

The Y-box binding protein 1 (YB-1; encoded by the *YBX1* gene) is a cold-shock, multifunctional DNA and RNA binding protein that regulates transcription, mRNA splicing, translation, and cellular stress responses (reviewed in [1,2,3,4]). YB-1 has been implicated in numerous oncogenic processes across multiple cancer types, including regulation of cell cycle progression from the G1/S transition through mitosis and cytokinesis [5,6,7,8,9,10,11,12,13,14]. It also plays a critical role in DNA damage repair, including mismatch repair and double-strand break repair, thereby enabling tolerance to replication stress [15,16,17,18,19].

In the context of epithelial–mesenchymal transition (EMT), YB-1 acts downstream of Twist to promote the expression of EMT markers such as Snail and vimentin [7,20,21,22,23]. In addition, YB-1 regulates mRNA splicing of key genes, including CD44, which promotes EMT-associated plasticity and invasion, and KLF5, which reinforces EMT-associated transcriptional programmes [24,25]. Effects on EMT are mediated in part through translational activation of HIF1α, a key regulator of hypoxic responses [26], modulation of IL-6 expression [27], and targeting of the intrinsic PD-1/PD-L1 pathway [28].

Furthermore, YB-1 contributes to therapeutic resistance through transcriptional regulation of multi-drug resistance transporters-1 (MDR-1) such as ABCB1 [29,30,31], activation of the MDM2/p53 axis [32,33], suppression of EGR1 expression [34], and activation of AKT signalling [35]. Clinically, elevated YB-1 expression and aberrant subcellular localization are consistently associated with aggressive disease and poor prognosis across a broad spectrum of malignancies [1,2,13,31], underscoring its central role in cancer biology.

A central conceptual question emerging from these observations is how a single gene such as *YBX1* can be implicated in such a broad spectrum of oncogenic phenotypes. Rather than independently driving each process, YB-1 may perturb one or more fundamental cellular programmes. In a context-dependent manner, these perturbations could result in diverse downstream consequences shaped by tumour lineage, microenvironmental stress, and co-occurring genetic alterations.

A potential explanation for these diverse phenotypic associations is that *YBX1* may be associated with fundamental cellular programmes linked to genomic instability-related phenotypes. Genomic instability encompasses both chromosomal alterations and the accumulation of somatic mutations, processes that can promote tumour evolution, phenotypic plasticity, metastatic competence, immune evasion, and therapeutic resistance (reviewed in [36,37,38,39,40]). These features arise through multiple mechanisms, including defects in DNA damage repair, replication stress, chromosome segregation, and cell cycle checkpoint control, and are often facilitated by loss of key tumour suppressors such as *TP53* [36,41]. Importantly, these processes may not be uniformly regulated across tumour types, reflecting the diverse biological contexts in which genomic instability emerges.

Genomic instability is closely intertwined with tumour hypoxia, a common feature of advanced tumours, with hypoxic stress both driving and resulting from genome destabilisation, thereby promoting the selection of aggressive tumour clones [38]. Although YB-1 has been experimentally linked to cell cycle defects, DNA damage responses, replication stress, and hypoxia-associated signalling [42], it remains unclear whether these processes converge to define a conserved YB-1-associated tumour state across cancers.

Recent studies have begun to examine *YBX1* in a pan-cancer context. The *YBX* gene family comprises three closely related Y-box-binding proteins, *YBX1*, *YBX2*, and *YBX3*, which share a conserved cold shock domain but exhibit distinct expression patterns and biological functions. Pan-cancer analyses of the *YBX* gene family have demonstrated widespread overexpression of *YBX1* across tumour types, along with associations with oncogenic signalling pathways, immune microenvironment features, and adverse clinical outcomes [43]. Additional large-scale survival analyses have confirmed that high-*YBX1* expression is associated with poor overall survival across multiple solid tumours, while other bioinformatic studies have linked *YBX1* expression to immune infiltration and metabolic pathway activity in selected cancers [44,45]. Collectively, these studies establish *YBX1* as a pan-cancer-relevant gene with prognostic significance.

However, to date, existing analyses have largely focused on differential expression and outcome correlations, leaving fundamental questions unresolved. It remains unclear whether *YBX1* is commonly altered at the genomic level or whether its oncogenic relevance is primarily driven by changes in gene dosage and expression. Moreover, despite extensive experimental evidence linking YB-1 to diverse cellular processes, it remains unclear whether elevated YB-1 expression reflects a conserved transcriptional programme across cancers, how it relates to genomic features including chromosomal alterations and mutation burden, and whether it marks clinically aggressive tumour states, either independently or in combination. The relationship between these states and genomic features, including chromosomal alterations and mutation burden, also remains unresolved.

In this study, we performed a comprehensive pan-cancer analysis across 53 independent cancer datasets using processed RNA-sequencing gene expression and matched somatic mutation data downloaded from cBioPortal [46,47,48]. We assessed the extent to which *YBX1* is altered at the genomic level across cancers and evaluated whether its oncogenic relevance is primarily driven by expression. We further examined whether elevated *YBX1* expression is associated with a conserved transcriptional programme across tumour types and investigated its relationship with genomic features, including mutation and chromosomal alteration burden. Given the intrinsic link between chromosomal alterations and cell division, we also evaluated the extent to which these associations are explained by proliferative activity and assessed their consistency across tumour types. Finally, the clinical relevance of *YBX1* expression was examined in relation to tumour stage, grade, hypoxia, and patient outcomes. In parallel, we assessed whether YB-1 protein abundance across 31 TCGA tumour types is similarly associated with genomic features, tumour hypoxia, and clinical parameters. Together, these analyses provide a comprehensive framework to define the molecular and clinical context of *YBX1* expression across human cancers.

## 2. Results

### 2.1. Somatic Alteration and Expression Patterns of YBX1 in Pan-Cancer Datasets

**Figure 1:**
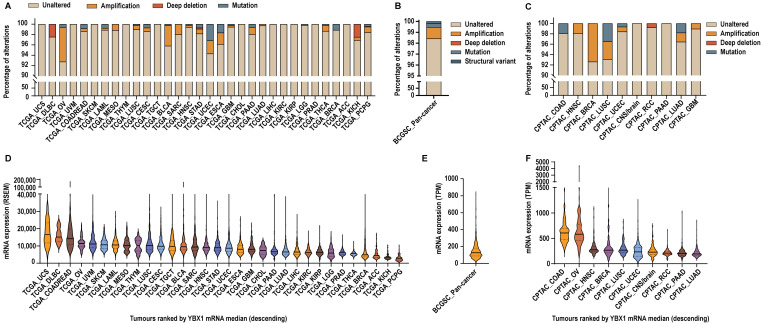
*YBX1* shows broad mRNA expression variability despite minimal genomic alterations across cancers. (**A**–**C**). Bar graphs showing the percentage of alterations in the *YBX1* gene from tumour samples within (**A**). Individual cancer types from the TCGA dataset, number of samples (*n*) = 10,198; (**B**). BCGSC pan-cancer study, *n* = 570; and (**C**). Individual cancer types from the CPTAC pan-cancer study, *n* = 1677. (**D**–**F**). Violin plots showing the distribution of *YBX1* mRNA expression across each study. Each violin represents the density of expression values within a tumour group, with the width indicating frequency. The median expression is shown as a dark solid horizontal line, and the interquartile range is indicated by dotted lines. (**D**). Individual studies from the TCGA dataset, *n* = 9367; (**E**). BCGSC pan-cancer study, *n* = 570; and (**F**). Individual studies from the CPTAC dataset, *n* = 1956.

Oncogenes are frequently driven by changes in copy number or mutations [22]. Therefore, we sought to determine whether *YBX1* follows a similar pattern across cancers. Analysis of multiple somatic pan-cancer datasets, including the TCGA, CPTAC, and BCGSC, encompassing over 12,000 samples across 33 tumour types from 53 studies, revealed that *YBX1* is largely unaltered, with amplifications occurring in only ~0.5–8% of cases and mutations in less than ~4% overall (Figure 1A–C and Figure S1).

A few tumour types exhibited slightly higher rates of alteration. In TCGA cohorts, *YBX1* was amplified in 6.65% of ovarian cancers (TCGA_OV), 3.93% of bladder cancers (TCGA_BLCA), 2.54% of endometrial cancers (TCGA_UCEC), and 2.2% of oesophageal cancers (TCGA_ESCA). Corresponding mutation frequencies were 3.13% in TCGA_UCEC and 1.65% in TCGA_ESCA cohorts. In CPTAC cohorts, amplification of *YBX1* was observed in 7.37% of breast cancers (CPTAC_BRCA), whereas no amplifications were reported in the corresponding TCGA_BRCA dataset. Additionally, ~3% of CPTAC_LUSC tumours harboured *YBX1* amplifications and another ~3% contained *YBX1* mutations, compared with less than 1% of altered cases in the TCGA_LUSC cohort. Despite these dataset- and tumour-specific differences, direct genetic alterations in *YBX1* are rare overall.

In contrast, pan-cancer transcriptomic analyses demonstrated that *YBX1* mRNA is broadly expressed across all tumour types but exhibits substantial inter-tumour heterogeneity within cohorts. To quantify this variability, we calculated the expression range within each dataset, defined as the ratio of the maximum to the minimum *YBX1* mRNA expression observed across tumours in that cohort. Across individual cancer types within the TCGA pan-cancer cohorts, *YBX1* mRNA expression ranges from 2.5-fold to 135-fold, with a median within-cohort expression range of 10.63-fold. The BCGSC pan-cancer cohort exhibited an expression range of 173-fold, while CPTAC studies showed expression ranges from 5.76-fold to 13,923-fold, with a median expression range of 12.3-fold across studies. These data highlight pronounced heterogeneity of *YBX1* mRNA expression within tumour cohorts, supporting the presence of distinct high-*YBX1*-expressing subsets across diverse cancer lineages (Figure 1D–F and Figure S1).

Collectively, these findings indicate that although *YBX1* is rarely altered at the genomic level, elevated and heterogeneous expression is a common feature across cancers, suggesting that its oncogenic relevance is primarily mediated through transcriptional or post-transcriptional mechanisms rather than frequent genetic alteration.

### 2.2. Pan-Cancer Correlation Analysis Reveals Conserved Transcriptional and Signalling Programmes Associated with YBX1 Across Molecular Levels

**Figure 2:**
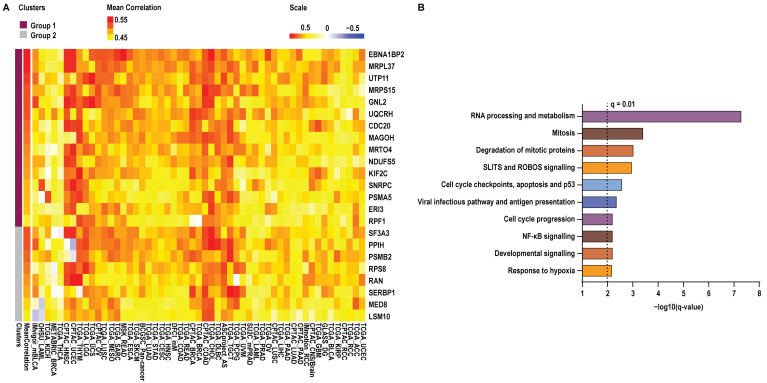
Genes positively correlated with *YBX1* mRNA expression and the top-enriched pathways. (**A**). Heatmap showing genes positively correlated with *YBX1* mRNA in at least 80% of the 53 datasets analysed (42/53). Significance was defined by Spearman’s correlation coefficient ≥ 0.3 and a *q*-value < 0.05. Group 1: Genes clustered in purple (upper quadrant) show exclusively positive correlations with *YBX1*. Group 2: Genes clustered in grey (lower quadrant) display negative correlations with *YBX1* in at least one dataset. Within each cluster, genes are ordered from top to bottom by descending mean correlation across all datasets. (**B**). Significantly enriched pathways (*q*-value ≤ 0.01) identified using EnrichR Reactome Pathway 2024 database for the genes positively correlated with *YBX1* mRNA across datasets.

To determine whether elevated *YBX1* mRNA expression is associated with specific cancer-related biological programmes, we performed Spearman’s correlation analyses between *YBX1* mRNA levels and the mRNA expression of 10,830 genes across 53 independent cancer datasets. Genes were selected based on consistent positive association with *YBX1* expression, defined as a Spearman correlation coefficient (ρ) ≥ 0.3 with q ≤ 0.05 in at least ~80% (42/53) of the datasets analysed. This analysis identified 23 genes meeting these criteria (Figure 2A).

Notably, 15 of these genes showed positive correlation across all 53 datasets, with significant positive correlations observed in at least 45 datasets, indicating a highly robust association with *YBX1* mRNA expression across diverse tumour contexts. The remaining eight genes were positively correlated in at least 51 of 53 datasets; among these, five genes (*PPIH*, *PSMB2*, *RPS8*, *RAN*, and *MED8*) were negatively correlated in a single dataset, while three genes (*SF3A3*, *SERBP1*, and *LSM10*) were negatively correlated in two datasets. For visualisation purposes, correlation coefficients across all datasets, including those not meeting significance thresholds, were displayed in the heatmap (Figure 2A).

In contrast, when the same consistency criteria were applied to negatively correlated genes, none were consistently shared across datasets (ρ ≤ −0.3, q ≤ 0.05 in ~80% of datasets), underscoring the specificity of positive *YBX1*-associated transcriptional programmes.

To assess the biological relevance of these associations, we next performed pathway enrichment analysis on genes whose mRNA expression correlated with *YBX1* mRNA levels. This revealed a strong overrepresentation of pathways involved in RNA processing and metabolism, cell cycle regulation at G1/S and G2/M, DNA replication and checkpoint control, hypoxia, and multiple oncogenic signalling pathways (Figure 2B, Supplementary Table S1). Collectively, these findings indicate that high-*YBX1* expression is tightly coupled to conserved proliferative and transcriptionally active tumour programmes across cancer types.

To extend these observations to the protein level, we analysed Reverse Phase Protein Array (RPPA) data from 31 TCGA tumour types to identify proteins whose abundance was significantly associated with YB-1 protein levels. Proteins were classified as positively or negatively associated based on a Spearman correlation coefficient (ρ ≥ 0.3 or ρ ≤ −0.3, respectively) with q ≤ 0.05 within each dataset. Unlike the mRNA analysis, protein-level associations were not consistently shared across tumour types at the level of individual proteins. Across the datasets, 27 tumour types contained significantly associated proteins to permit downstream analysis. Pathway enrichment analysis identified 283 positively associated and 163 negatively associated pathways, which were subsequently integrated by assessing their recurrence across tumour types (Supplementary Figure S2A,B and Supplementary Table S2).

Proteins positively associated with YB-1 were enriched in signalling-related pathways, including receptor tyrosine kinase signalling, cytokine and interleukin signalling, MAPK cascades, and cellular responses to external stimuli, reflecting active oncogenic signalling and microenvironmental responsiveness. In contrast, proteins negatively associated with YB-1 were enriched for pathways related to transcriptional regulation, DNA repair, apoptosis, and cell-cycle checkpoint control, including G2/M DNA damage checkpoint regulation and *TP53*-mediated transcriptional programmes.

Given that none of the genes consistently correlated with *YBX1* mRNA expression were represented in the RPPA dataset, direct comparison between transcript- and protein-level candidates at the gene level was not possible. Moreover, as no genes were consistently negatively correlated with *YBX1* mRNA across tumour types, cross-layer pathway integration was restricted to positively associated features. We therefore intersected pathways enriched from positively *YBX1*-associated mRNA expression with those derived from positively YB-1-associated protein abundance and assessed their recurrence across tumour types. This analysis identified a set of shared pathways consistently enriched across datasets (Supplementary Figure S2C), including transcriptional regulation (e.g., RNA polymerase II-mediated transcription and RUNX-dependent programmes), core gene expression processes, and cell-cycle-associated pathways such as G1/S transition, S-phase progression, cyclin-dependent kinase activity, and cell-cycle checkpoints. Additional enrichment was observed for apoptosis, cellular stress response pathways, developmental signalling pathways (including WNT and NOTCH), and pathways related to mRNA stability and post-transcriptional regulation.

Although several high-level biological processes, including cell-cycle regulation and apoptosis, were represented in both positively and negatively associated pathway sets, these corresponded to distinct components within those processes. Positively associated pathways were enriched for features linked to cell-cycle progression and signalling activity, whereas negatively associated pathways were enriched for DNA damage response and checkpoint-related processes (Supplementary Table S2).

Overall, these findings indicate that *YBX1* expression is associated with coordinated transcriptional, cell-cycle, and signalling-related programmes across cancer types.

### 2.3. Pan-Cancer Genomic and Pathway Correlates of YBX1 mRNA Expression

**Figure 3:**
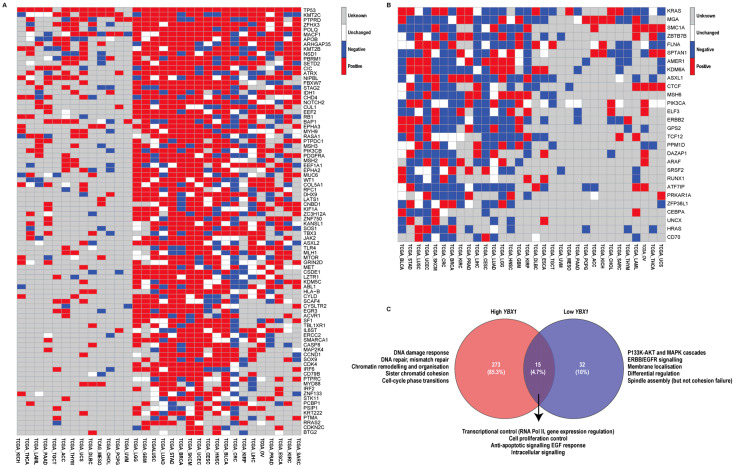
High- and low-*YBX1* tumours display distinct mutation frequencies in known driver genes. (**A**,**B**). Heatmaps showing known cancer driver genes that are frequently mutated in (**A**). high-*YBX1*, (**B**). low-*YBX1* tumours across all 32 tumour types in the TCGA dataset. Tumours were stratified into high- and low-*YBX1* groups based on the 75th and 25th percentiles of *YBX1* mRNA expression within each tumour type. For each gene, mutation enrichment was classified as positive (red, Δ > 0) if the mutation rate was higher, negative (blue, Δ < 0) if lower, and unchanged (white, Δ = 0) if no difference was observed in the high-*YBX1* group. Missing data are indicated in grey. Genes were ordered by the number of tumour types with positive enrichment, and hierarchical clustering was applied to both genes and tumour types to reveal patterns of mutation enrichment across datasets. (**C**). Venn diagram showing independent and overlapping pathways enriched in the EnrichR GO Biological Processes 2025 database (q-value ≤ 0.01) for genes frequently mutated in high-*YBX1*, low-*YBX1*, and shared tumour groups. The major functional classes of enriched GO Biological Processes are shown alongside each group, with full pathway details provided in Supplementary Table S3.

To investigate whether *YBX1* mRNA expression is associated with specific mutational patterns, we focused our analysis on the TCGA datasets, as they provide the most comprehensive, high-quality, and uniformly processed mutation and expression data across diverse cancer types. Mutation rates for each gene were compared between the high- and low-expression groups, and a delta value (Δ = high − low) was computed to quantify enrichment of mutations in high-*YBX1* tumours. Genes were then classified as positive (Δ > 0), negative (Δ < 0), or unchanged (Δ = 0), and the number of tumour types with positive (pos) versus negative (neg) enrichment was summarised as a pos/neg ratio. Genes with a pos/neg ratio > 2 were considered consistently enriched in high-*YBX1* tumours, whereas those with a ratio ≤ 0.8 were depleted (Figure 3A,B).

Using this approach, *TP53*, the key tumour suppressor and “guardian of the genome”, was consistently mutated at a higher frequency in high-*YBX1* tumours in 29 of the 32 TCGA datasets analysed (Figure 3A). Beyond *TP53*, multiple genes with well-established roles in maintaining genome integrity were preferentially mutated in high-*YBX1* tumours (Figure 3A). *TP53* was mutated in all datasets where data were available, while the remaining genes were mutated across most datasets. These genes function across multiple genome-stabilising processes, including DNA repair (*POLQ*, *SETD2*, *ATRX*, *MSH2*, *MSH3*, *MLH1*, *ERCC2*, *RFC1*, *DHX9*, *PSIP1*), chromatin regulation (*KMT2C*, *KMT2B*, *NSD1*, *PBRM1*, *CHD4*, *KANSL1*, *KDM5C*, *ASXL2*, *SMARCA1*, *SCAF4*), chromosome segregation (*STAG2*, *NIPBL, RB1*, *LATS1*, *FBXW7*, *CUL1*), and cell-cycle checkpoint control (*CDK4*, *CCND1*, *CDKN2C*). Collectively, mutations in these pathways are known to promote genomic instability through defects in DNA damage repair, chromatin organisation, and mitotic fidelity.

In contrast, low-*YBX1* tumours (Figure 3B) were enriched for mutations in receptor-proximal signalling and lineage-associated genes, including *KRAS*, *ERBB2*, *PIK3CA*, *ARAF*, *HRAS*, *ELF3*, and *CEBPA*. Compared with high-*YBX1* tumours, this pattern was less uniform across datasets, but is consistent with a more signal-driven, differentiation-associated tumour state (Figure 3B).

To extend these observations beyond individual genes, we performed gene ontology-based pathway enrichment analysis using cancer driver genes differentially mutated between high- and low-*YBX1* tumours. While both groups shared enrichment for core oncogenic programmes related to transcriptional regulation, cell proliferation, and growth factor responsiveness, high-*YBX1* tumours were enriched for pathways associated with DNA damage response, DNA repair, mismatch repair, chromatin remodelling, chromosome organisation, sister chromatid cohesion, and cell-cycle phase transitions (Figure 3C, Supplementary Table S3). These pathway-level signatures are characteristic of replication stress and chromosomal instability, providing functional support for the elevated frequency of genome maintenance gene mutations observed in high-*YBX1* tumours. Conversely, pathways uniquely enriched in low-*YBX1* tumours were largely restricted to PI3K–AKT, MAPK, and ERBB/EGFR signalling cascades, as well as differentiation-related processes. Together, these findings are consistent with high-*YBX1* tumours exhibiting greater proliferative and transcriptionally plastic features alongside increased enrichment of genomic instability-related processes.

### 2.4. YBX1 Expression Is Associated with Genomic Instability-Related and Hypoxia-Associated Tumour Features

To determine whether elevated *YBX1* mRNA expression is associated with increased genomic instability, we next examined whether high-*YBX1* tumours exhibit increased mutational burden, copy number alterations, or defects in homologous recombination (HR), a high-fidelity DNA repair pathway. Tumours were stratified based on *YBX1* mRNA expression, with high-*YBX1* tumours defined as those at or above the 75th percentile and low-*YBX1* tumours defined as those at or below the 25th percentile.

**Figure 4:**
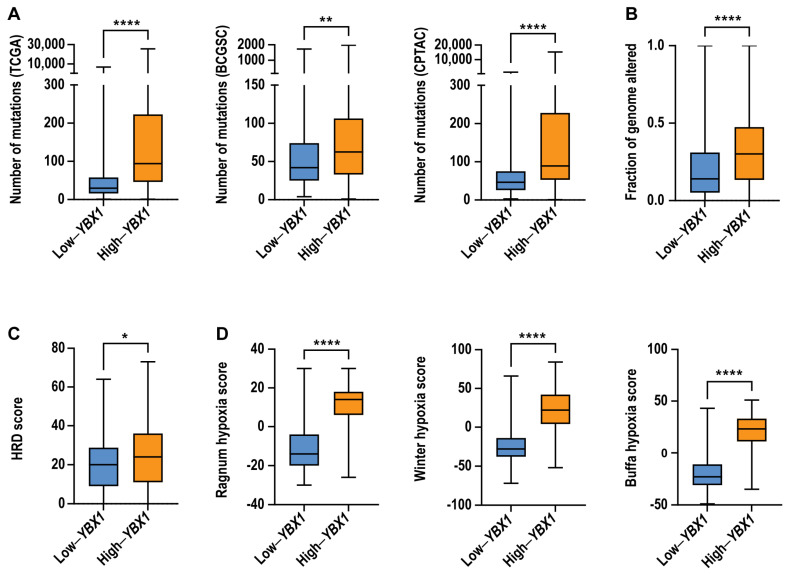
High-*YBX1* tumours display increased genomic instability and a higher hypoxia score. Box and whiskers plots comparing the distribution of (**A**). total mutation counts in high- and low-*YBX1* tumours across the TCGA (high-*YBX1*, n = 2360; low-*YBX1*, n = 2393; **** *p* < 0.0001), BCGSC (high-*YBX1*, n = 142; low-*YBX1*, n = 143; ** *p* = 0.0015), and CPTAC (high-*YBX1*, n = 483; low-*YBX1*, n = 278; **** *p* < 0.0001) datasets. Significance: Mann–Whitney U test, *p* < 0.05 is considered statistically significant. (**B**). FGA in high-*YBX1* (n = 2360) and low-*YBX1* (n = 2393) tumours in the TCGA dataset (**** *p* < 0.0001). Significance: Mann–Whitney U test, *p* < 0.05 is considered statistically significant. (**C**). HRD score in high-*YBX1* (n = 144) and low-*YBX1*(n = 143) tumours in the BCGSC dataset (* *p* = 0.0145). Significance: Mann–Whitney U test, *p* < 0.05 is considered statistically significant. (**D**). Hypoxia scores in high-*YBX1* (n = 1722) and low-*YBX1* (n = 2171) tumours in the TCGA dataset (Ragnum: **** *p* < 0.0001; Buffa: **** *p* < 0.0001; and Winter: **** *p* < 0.0001). Significance: Wilcoxon test, *p* < 0.05 is considered statistically significant. (**A**–**D**). The central line represents the median, the box indicates the interquartile range (25th–75th percentile), and the whiskers extend to the most extreme data point within this range.

Across the TCGA pan-cancer cohort, as well as independent BCGSC and CPTAC datasets, tumours with high-*YBX1* expression exhibited significantly higher numbers of somatic mutations compared to low-*YBX1* tumours (Figure 4A). In addition, high-*YBX1* tumours displayed increased chromosomal alteration burden, as measured by fraction of genome altered (FGA), within the TCGA cohort (Figure 4B). Consistent with these findings, high-*YBX1* tumours in the BCGSC dataset were more likely to exhibit homologous recombination deficiency (Figure 4C). Furthermore, high-*YBX1* tumours were associated with significantly elevated hypoxia scores across multiple hypoxia signatures (Figure 4D).

To assess whether these associations were also observed at the protein level, we analysed Reverse Phase Protein Array (RPPA) data from 31 TCGA tumour types. Tumours were stratified based on YB-1 protein abundance using the same percentile thresholds (≥75th percentile, high; ≤25th percentile, low). Consistent with the mRNA-based analysis, tumours with high YB-1 protein levels were associated with increased mutation counts, a higher fraction of the genome altered, and elevated hypoxia scores (Supplementary Figure S3A–C).

Together, these findings indicate that elevated *YBX1* expression, at both the mRNA and protein levels, is associated with tumour features linked to increased mutational burden, genomic alteration, and hypoxic tumour microenvironments across cancer types.

### 2.5. Association Between YBX1 mRNA Expression and Genomic Instability-Related Features Is Influenced by Proliferative Activity

Given the observed association between *YBX1* mRNA expression and genomic instability-related features, we next assessed the extent to which these relationships are influenced by proliferative activity. *YBX1* mRNA expression was moderately correlated with proliferation, as measured by a multi-gene G2/M checkpoint score (ρ = 0.53, *p* < 0.0001), indicating overlap with cell cycle-associated transcriptional programmes. To distinguish general proliferative effects from mitosis-associated processes, we compared two proliferation metrics: MKI67, a marker of overall proliferative activity, and a multi-gene G2/M checkpoint score, which captures transcriptional programmes associated with the G2/M phase of the cell cycle. This distinction enables the separation of general proliferation from mitosis-associated processes linked to chromosomal segregation and instability.

**Figure 5:**
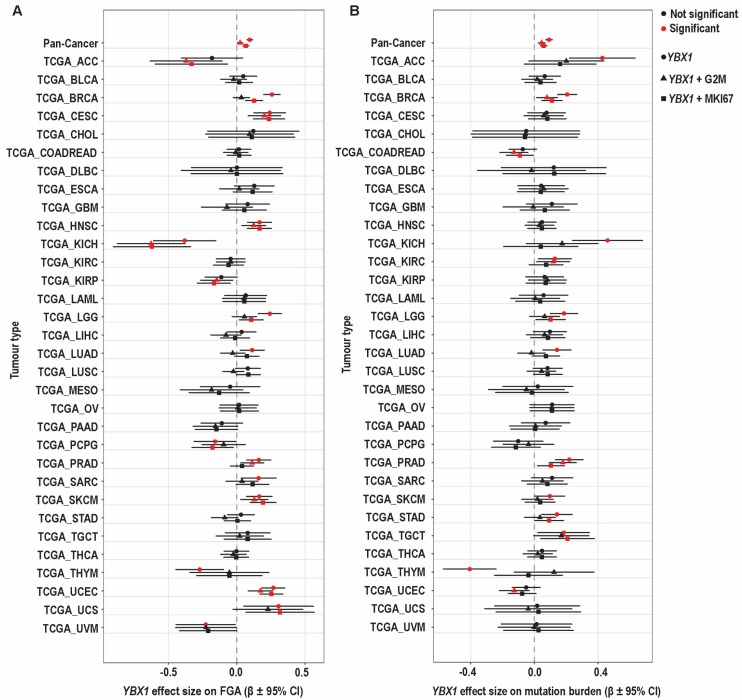
Differential effects of proliferation adjustment on *YBX1* associations with chromosomal alterations and mutation burden. Forest plots showing the association between *YBX1* mRNA expression and (**A**). Fraction of genome altered (FGA) and (**B**). Mutation burden across tumour types. For each tumour type, three models are shown: unadjusted (*YBX1* alone), adjusted for MKI67, and adjusted for the G2/M (G2M) checkpoint score. Each point represents the estimated effect size (β) of *YBX1* expression within a tumour type, with horizontal lines indicating 95% confidence intervals. The dashed vertical line indicates no effect (β = 0). The pan-cancer estimate is shown at the top of each panel. Red points indicate statistically significant associations (*p* < 0.05), while black points indicate non-significant results. Different point shapes denote the model used (unadjusted, MKI67-adjusted, or G2M-adjusted). Linear regression models were used to estimate associations within each tumour type and at the pan-cancer level. Adjustment for MKI67 reflects general proliferative activity, whereas adjustment for the G2M checkpoint score captures mitotic and G2/M phase-associated processes. A *p* < 0.05 was considered statistically significant.

Using these proliferation metrics, we next examined the relationship between *YBX1* mRNA expression and genomic instability across tumour types. At the pan-cancer level, *YBX1* mRNA expression was associated with both FGA and mutation burden. Adjustment for proliferation attenuated these associations, with a greater reduction observed following adjustment for the G2/M checkpoint score compared to MKI67 (Figure 5 and Table 1).

At the individual tumour-type level, the relationship between *YBX1* mRNA expression and FGA was heterogeneous but broadly consistent with the pan-cancer pattern (Figure 5A). Several tumour types, including CESC, HNSC, SKCM, and UCEC, showed positive associations that remained evident after adjustment. In BRCA and LGG, associations persisted after adjustment for MKI67 but were attenuated following adjustment for G2M, indicating a strong contribution from mitotic processes. In LUAD, the association was present only in unadjusted models and was attenuated after adjustment, whereas in PRAD, the association remained significant after adjustment for G2/M but not MKI67. KICH showed a consistent negative association across models, while THYM, UVM, and PCPG showed negative or model-dependent effects.

In many tumour types, including BLCA, CHOL, COADREAD, DLBC, ESCA, GBM, KIRC, LAML, LIHC, LUSC, MESO, OV, PAAD, STAD, TGCT, and THCA, no statistically significant association between *YBX1* and FGA was observed. However, directional trends were evident: CHOL, ESCA, LAML, and TGCT showed consistently positive effect estimates, whereas KIRC, MESO, and PAAD demonstrated predominantly negative trends. The remaining tumour types showed effect estimates centred near zero.

Across tumour types, adjustment for proliferation reduced the magnitude of the *YBX1*–FGA association, with a greater effect observed for G2/M than MKI67, while preserving the direction of association. This pattern indicates that the *YBX1*–FGA relationship reflects shared mitotic and cell cycle–associated biology, consistent with the role of G2/M progression in generating chromosomal instability.

In contrast, the association between *YBX1* mRNA expression and mutation burden was observed in fewer tumour types and was less consistent (Figure 5B). Positive associations were detected in ACC, KICH, LGG, LUAD, and SKCM in unadjusted models but were attenuated after adjustment. In BRCA and PRAD, associations persisted after adjustment, while in STAD, LGG, and TGCT, associations were retained after adjustment for MKI67 but not G2M. THYM showed a negative association in unadjusted models that was lost after adjustment. Among tumour types without statistically significant associations, effect estimates were generally weak and inconsistently directed, with some showing positive trends and others centred near zero.

Taken together, these findings show that *YBX1* expression is associated with both chromosomal and mutational measures of genomic instability, with associations involving FGA observed more broadly across tumour types. The attenuation observed following adjustment for proliferation, particularly with the G2M checkpoint score, suggests that these relationships are influenced by mitotic and cell cycle-associated processes, whereas the association with mutation burden is more variable and less consistently affected by proliferation.

Overall, *YBX1* mRNA expression is associated with tumour features linked to chromosomal instability, with these relationships largely influenced by proliferative and mitotic processes, particularly for chromosomal alteration burden. The attenuation observed following adjustment for proliferation, especially with G2/M-associated transcriptional programmes, indicates that these associations reflect shared cell-cycle and mitotic biology. Taken together, these findings indicate that, at the transcriptomic level, *YBX1* mRNA expression does not independently capture chromosomal instability, but instead marks a proliferative tumour state in which such features arise.

### 2.6. High-YBX1 Expression Is Associated with Advanced Tumour Stage, Higher Grade, and Poor Clinical Outcomes

To determine whether the genomic and transcriptional features associated with high-*YBX1* expression are reflected in clinical behaviour, we next examined the relationship between *YBX1* expression and key clinicopathological parameters used in patient management. Tumours were stratified into high- and low-*YBX1* groups based on *YBX1* mRNA expression (high ≥ 75th and low ≤ 25th percentiles, respectively), and analyses were performed using clinical annotations available from the pan-cancer TCGA dataset.

**Figure 6:**
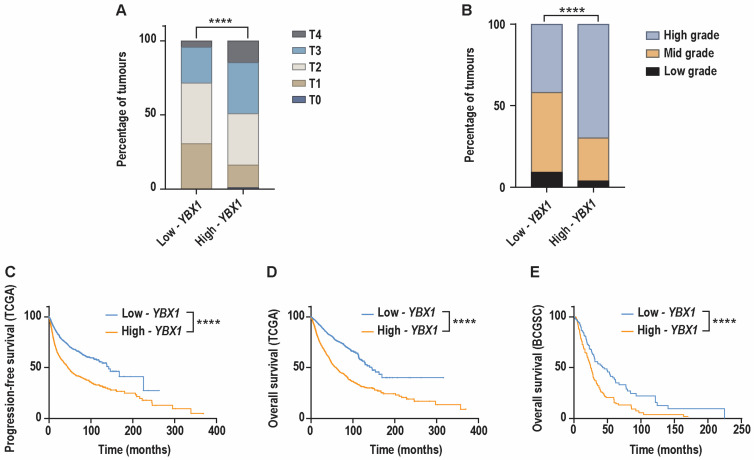
Patients with high-*YBX1* mRNA expression exhibit more advanced tumour stage and grade, with reduced survival. Bar graph comparing the percentage distribution of (**A**). AJCC tumour stage in patients with high-*YBX1* (n = 1569) and low-*YBX1* (n = 1987) tumours across the TCGA dataset (**** *p* < 0.0001). Significance: Chi-squared test, *p* < 0.05 is considered statistically significant. T0: primary tumour not found or cannot be measured; T1-T4: increasing in tumour size and invasion into nearby tissues. (**B**). Neoplasm histologic grade in patients with high-*YBX1* (n = 1027) and low-*YBX1* (n = 684) tumours across the TCGA dataset (**** *p* < 0.0001). Significance: Chi-squared test, *p* < 0.05 is considered statistically significant. Low grade: well-differentiated tumour cells; mid grade: moderately differentiated tumour cells; high grade: poorly differentiated to undifferentiated tumour cells. (**C**–**E**). Kaplan–Meier plots comparing (**C**). progression-free survival in the TCGA dataset (high-*YBX1* (n = 2399) and low-*YBX1* (n = 2511)); (**D**). overall survival of patients in the TCGA dataset (high-*YBX1* (n = 2481) and low-*YBX1* (n = 2514)); and (**E**). overall survival of patients in the BCGSC dataset (high-*YBX1* (n = 143) and low-*YBX1* (n = 144)). (**C**–**E**): Significance: log-rank test, *p* < 0.05 is considered statistically significant, **** *p* < 0.0001.

Consistent with the proliferative and genomic features associated with high-*YBX1* expression, we found that these tumours were significantly enriched for advanced disease stage. Specifically, high-*YBX1* tumours exhibited a significantly higher proportion of AJCC T3- and T4-stage disease compared to low-*YBX1* tumours (Figure 6A). In parallel, histopathological assessment revealed that histologic grade was also significantly higher in high-*YBX1* tumours relative to low-*YBX1* tumours, further supporting a more aggressive tumour phenotype (Figure 6B).

We next evaluated whether elevated *YBX1* mRNA expression was associated with earlier disease recurrence. Progression-free survival was available for the pan-cancer TCGA cohort. This analysis demonstrated that patients with high-*YBX1* tumours experienced a significantly shorter progression-free survival compared to those with low-*YBX1* tumours (Figure 6C).

We next evaluated the association between *YBX1* mRNA expression and clinical outcomes. In the TCGA pan-cancer cohort, patients with high-*YBX1* tumours had significantly shorter progression-free survival compared to those with low-*YBX1* tumours (Figure 6C). Similarly, analysis of overall survival in both the TCGA cohort and the metastatic pan-cancer BCGSC dataset showed that high-*YBX1* expression was associated with reduced overall survival (Figure 6D,E).

To assess whether these associations were also observed at the protein level, we analysed YB-1 protein abundance using RPPA data across 31 TCGA tumour types. Tumours were stratified using the same percentile thresholds (≥75th and ≤25th percentiles). Consistent with the mRNA-based analysis, tumours with high YB-1 protein levels were enriched for advanced tumour stage (Supplementary Figure S4A). A similar trend was observed for histologic grade (Supplementary Figure S4B), although this did not reach statistical significance. In addition, patients with tumours expressing high levels of YB-1 protein showed reduced overall survival compared to those with low protein expression (Supplementary Figure S4C).

Taken together, these findings show that elevated *YBX1* expression at both the mRNA and protein levels is associated with clinicopathological features and outcomes indicative of more aggressive disease.

## 3. Discussion

This study provides comprehensive pan-cancer integration of genomic, transcriptomic, proteomic, and clinical data to characterise tumour features associated with elevated *YBX1* expression across multiple datasets and tumour types. Through this integrative analysis, we identify a reproducible set of molecular features that co-occur with high *YBX1* expression, encompassing cell cycle and mitotic programmes, RNA processing, signalling pathways, and features related to DNA damage response and genome maintenance, alongside clinicopathological characteristics indicative of more advanced disease. Although these analyses are necessarily based on bulk datasets and are therefore subject to batch effects [49], variability in sequencing platforms [50,51], clinical annotation [52], and expression-based stratification across tumour types, the consistent emergence of these patterns across independent cohorts supports their reproducibility and relevance at the level of pan-cancer association.

The observed transcriptomic and proteomic associations are consistent with known roles of YB-1 in cell cycle regulation, RNA processing, and genome maintenance. Previous studies have shown that YB-1 regulates E2F-driven transcriptional programmes associated with tumour growth [13], promotes cell cycle progression via CDC6-dependent pathways [5], and modulates the G2/M transition through regulation of cyclins, including cyclin A1 [12], while also influencing G2/M arrest through interactions with cyclin D1 [11] and contributing to mitotic and cytokinetic regulation [6,9]. Concordant with these established functions, transcriptomic and proteomic analyses in the present study demonstrate consistent enrichment of cell cycle-related pathways in tumours with high *YBX1* expression across tumour types. In addition to associations with cell cycle checkpoint pathways, proteomic analyses also identified positive associations with signalling pathways, including MAPK, receptor tyrosine kinase, and cytokine/interleukin signalling pathways that are closely linked to proliferative capacity.

Tumours with high *YBX1* expression also exhibit features associated with altered genome maintenance. Genomic analyses demonstrate enrichment for mutations in *TP53*, as well as mutations affecting genes involved in DNA repair, chromatin organisation, chromosome segregation, and cell cycle progression, processes central to chromosomal alteration. *TP53* is a key regulator of genomic integrity through its control of cell cycle arrest, DNA repair, and apoptosis, and disruption of *TP53* is strongly associated with genomic instability, including both chromosomal alterations and mutational accumulation [53]. Proteomic analyses further show reduced association with *TP53*-mediated transcription, DNA repair, and cell cycle checkpoint pathways, consistent with a relative reduction in genome surveillance pathway engagement in high-*YBX1* tumours. In line with this, prior studies have reported that YB-1 can antagonise p53-mediated tumour suppression through activation of MDM2 and repression of p53 target genes [32,33], providing a potential mechanistic context for the inverse association observed between *YBX1* expression and *TP53* pathway activity. Additional support for links between YB-1 and genome maintenance comes from studies demonstrating interactions between YB-1 and DNA repair machinery [15,16,17], promotion of double-strand break repair via DNA-PK-dependent mechanisms [16], and disruption of mismatch repair through interactions with PCNA and MutSα [19].

Despite these associations, the relationship between *YBX1* expression and mutation burden is heterogeneous across tumour types. Although tumours with high *YBX1* expression exhibit increased mutation burden at the pan-cancer level, this association varies across individual tumour contexts, suggesting that mutational processes are influenced by tumour-specific factors rather than by *YBX1* expression alone. This variability is consistent with the well-established diversity of mutational mechanisms in cancer, including mismatch repair deficiency, APOBEC activity, and environmental exposures, which differ markedly across tumour lineages [54]. Consequently, the association between *YBX1* expression and mutation burden appears context-dependent and is not uniformly coupled to broader genome maintenance-related features.

At both the transcriptomic and proteomic levels, YB-1 expression is also associated with developmental signalling pathways, including WNT and NOTCH, RUNX2-associated transcriptional programmes, and altered RNA processing and metabolic pathways. These features are consistent with a more plastic, stemness-associated tumour context that may favour invasion, adaptation, and therapeutic resistance. These pathway-level associations are supported by prior studies implicating *YBX1* in transcriptional regulation and signalling through NOTCH [55] and WNT pathways [56,57]. In addition to its roles in cell cycle regulation, YB-1 functions as a multifunctional RNA-binding protein involved in RNA processing, stability, and metabolism, with studies demonstrating binding to A/C-rich RNA sequences to promote RNA stability and alternative splicing [58,59,60]. Enrichment of RUNX2- and RUNX3-associated transcriptional programmes further aligns with tumour contexts associated with proliferation, invasion, and EMT [7,21,22,26,27,28].

Overall, these findings are consistent with tumour contexts in which reduced engagement of checkpoint and genome maintenance pathways co-occurs with sustained proliferative and signalling activity. In line with these observations, high-*YBX1* tumours are enriched for advanced disease stage and higher histologic grade and are associated with poorer clinical outcomes at both the mRNA and protein levels, although associations at the protein level appear more variable across tumour contexts. These findings are consistent with previous reports linking elevated *YBX1* expression to poor prognosis [1,2,45,61], therapy resistance [29,30,31,32,34,35,56], and epithelial–mesenchymal transition (EMT) across cancers [7,20,21,22,23].

Taken together, this pan-cancer analysis places prior observations regarding YB-1 in a broader, integrative context. While YB-1 has been linked to proliferation, mitotic regulation, chromosomal instability, and DNA repair in individual experimental systems, our results indicate that across tumour types, *YBX1* expression primarily marks proliferative and mitotic tumour states that are associated with, rather than independently predictive of, genomic instability-related features. Notably, the attenuation of associations with chromosomal alteration burden following adjustment for proliferative activity supports the interpretation that these relationships are embedded within shared cell-cycle and mitotic processes rather than representing a distinct *YBX1*-defined genomic instability programme.

These findings should also be interpreted in the context of several study limitations. The analyses presented here are correlative and do not establish causal relationships between *YBX1* expression and the molecular or clinical features identified. In addition, although pan-cancer consistency was observed across multiple datasets, residual confounding by tumour lineage and molecular subtype remains possible, particularly given the known heterogeneity within tumour types. Furthermore, the use of bulk transcriptomic and proteomic datasets limits the resolution of cell-state and microenvironmental contributions to *YBX1*-associated programmes.

Within these limitations, the present study provides a framework for future investigation into the molecular mechanisms underlying these associations and their clinical relevance. Such studies will require functional validation using inducible and CRISPR-based perturbation models, organoid and in vivo systems, and cell cycle–resolved proteomic approaches to delineate YB-1-dependent regulatory networks. Longitudinal analyses integrating genomic profiling with DNA damage and repair assays will also be necessary to determine how *YBX1* expression relates to mutation accumulation and tumour evolution in specific biological contexts. Defining these context-specific dependencies will be critical for assessing whether *YBX1* expression can serve as a clinically meaningful biomarker for patient stratification or therapeutic targeting.

## 4. Materials and Methods

### 4.1. Data Retrieval from the cBioPortal Database

A total of 53 datasets from cBioPortal (accessed in January 2026) [46,47,48] were downloaded and integrated to analyse the genomic, transcriptomic, and clinical characteristics associated with *YBX1* mRNA. This included two pan-cancer datasets, the British Columbia Genome Sciences Centre Pan-cancer Analysis of Advanced and Metastatic Tumors (BCGSC, 2020) and the Cancer Genome Atlas PanCancer Atlas studies (TCGA, PanCancer Atlas); 16 studies of various cancer types harmonised by the Clinical Proteomic Tumor Analysis Consortium (CPTAC), including colon adenocarcinoma (CPTAC, 2025), colon cancer (CPTAC-2 Prospective, 2019), CNS/brain cancer (CPTAC, 2025), glioblastoma (CPTAC, 2021), head and neck carcinoma, other (CPTAC, 2025), renal cell carcinoma (CPTAC, 2025), lung adenocarcinoma (CPTAC, 2020; CPTAC, 2025), lung squamous cell carcinoma (CPTAC, 2021; CPTAC, 2025), ovarian cancer (CPTAC, 2025), pancreatic cancer (CPTAC, 2025), endometrial carcinoma (CPTAC, 2020), Uterine endometrioid carcinoma (CPTAC, 2025), breast cancer (CPTAC, 2025), proteogenomic landscape of breast cancer (CPTAC, 2020); and 9 miscellaneous studies of various cancer types including metastatic bladder urothelial carcinoma (IMvigor210 Phase II Trial, 2024), renal cell carcinoma (IMmotion150 Clinical Trial, 2018), metastatic melanoma (DFCI, 2019), rectal cancer (MSK, 2022), breast cancer (METABRIC, 2012 and 2016), Diffuse Glioma (GLASS Consortium), acute myeloid leukaemia (OHSU, 2018), metastatic prostate adenocarcinoma (SU2C/PCF Dream Team, 2019), and the Angiosarcoma Project–Count Me In (Provisional, April 2025).

The BCGSC dataset (referred to as BCGSC, 2020) was retrieved at the pan-cancer level.

The TCGA PanCancer Atlas dataset (referred to as TCGA) was retrieved both at the pan-cancer level and across 33 individual cancer types. These are uterine carcinosarcoma (TCGA_UCS), diffuse large B-cell lymphoma (TCGA_DLBC), ovarian serous cystadenocarcinoma (TCGA_OV), uveal melanoma (TCGA_UVM), skin cutaneous melanoma (TCGA_SKCM), acute myeloid leukaemia (TCGA_LAML), colorectal adenocarcinoma (TCGA_COADREAD; comprising colon adenocarcinoma (TCGA_COAD) and rectal adenocarcinoma (TCGA_READ)), mesothelioma (TCGA_MESO), thymoma (TCGA_THYM), lung squamous cell carcinoma (TCGA_LUSC), cervical squamous cell carcinoma (TCGA_CESC), testicular germ cell tumours (TCGA_TGCT), bladder urothelial carcinoma (TCGA_BLCA), sarcoma (TCGA_SARC), head and neck squamous cell carcinoma (TCGA_HNSC), stomach adenocarcinoma (TCGA_STAD), uterine corpus endometrial carcinoma (TCGA_UCEC), esophageal adenocarcinoma (TCGA_ESCA), glioblastoma multiforme (TCGA_GBM), cholangiocarcinoma (TCGA_CHOL), pancreatic adenocarcinoma (TCGA_PAAD), lung adenocarcinoma (TCGA_LUAD), liver hepatocellular carcinoma (TCGA_LIHC), kidney renal clear cell carcinoma (TCGA_KIRC), kidney renal papillary cell carcinoma (TCGA_KIRP), brain lower grade glioma (TCGA_LGG), prostate adenocarcinoma (TCGA_PRAD), thyroid carcinoma (TCGA_THCA), breast invasive carcinoma (TCGA_BRCA), adrenocortical carcinoma (TCGA_ACC), kidney chromophobe (TCGA_KICH), and pheochromocytoma and paraganglioma (TCGA_PCPG).

Data from 16 CPTAC-harmonised studies (referred to as CPTAC) across 11 individual cancer types were retrieved, including colon cancer (CPTAC_COAD), ovarian cancer (CPTAC_OV), head and neck carcinoma (CPTAC_HNSC), breast cancer (CPTAC_BRCA), lung squamous cell carcinoma (CPTAC_LUSC), endometrial carcinoma (CPTAC_UCEC), CNS/brain Cancer (CPTAC_CNS/brain), renal cell carcinoma (CPTAC_RCC), pancreatic cancer (CPTAC_PAAD), lung adenocarcinoma (CPTAC_LUAD), and glioblastoma (CPTAC_GBM).

Data from 9 miscellaneous studies across 9 individual cancer types were retrieved, including metastatic bladder urothelial carcinoma (IMvigor_mBLCA), Renal Cell Carcinoma (IMmotion_RCC), metastatic melanoma (DFCI_mM), rectal adenocarcinoma (MSK_READ), breast cancer (METABRIC_BRCA), diffuse glioma (GLASS_DG), Acute myeloid leukaemia (OHSU_LAML), metastatic prostate adenocarcinoma (SU2C_mPRAD), and angiosarcoma (ASCproject_AS).

### 4.2. Genomic and Transcriptomic Profiles of YBX1

Genomic and transcriptomic profiles of *YBX1*, along with their corresponding tumour origins, were retrieved by querying the *YBX1* gene in cBioPortal across all datasets.

Genomic alterations assessed include mutations (missense, nonsense, truncation, frameshift), copy number variations (amplifications and deletions), and structural variations. Mutation and copy number variation data were available for all BCGSC, TCGA, and CPTAC datasets, whereas structural variation data were only available in the BCGSC and TCGA datasets. For genomic alterations, the BCGSC dataset was assessed at the pan-cancer level, the TCGA dataset was assessed across 33 individual cancer types, the CPTAC dataset was assessed across 10 out of 11 individual cancer types due to data availability, and the 9 miscellaneous studies were assessed individually (Table 2).

The mRNA expression of *YBX1* analysed in this study was determined by RNA sequencing across all studies, except for the METABRIC_BRCA dataset, which was profiled using microarray. For *YBX1* mRNA expression, the BCGSC dataset was assessed at the pan-cancer level, the TCGA dataset was assessed across 32 individual cancer types, the CPTAC dataset was assessed across 10 out of 11 individual cancer types due to data availability, and the 9 miscellaneous studies were assessed individually (Table 2).

Statistical analyses and graphical representations were conducted using GraphPad Prism (version 11.0.0) and Adobe Illustrator (version 29.5.1 (64 bit)).

### 4.3. Spearman’s Correlation Analysis and Pathway Analysis

The mRNA co-expression data against *YBX1* mRNA was downloaded from cBioPortal by querying the *YBX1* gene across the BCGSC pan-cancer dataset, the TCGA pan-cancer dataset, the CPTAC dataset, and across the 9 miscellaneous studies. The sample sizes for each dataset are detailed in Table 2.

To identify genes consistently correlated with *YBX1* expression across multiple datasets, we collected all available CSV files containing gene-level correlation data (Spearman’s correlation coefficients and associated q-values) between *YBX1 mRNA* and other genes across independent cancer datasets. Data were imported and standardised using R (version 4.4.3) with the dplyr, tidyr, tibble, and pheatmap packages. Columns were harmonised across datasets to include gene, cytoband, correlation (ρ), *p*-value (*p*), q-value (q), and dataset identifiers. Only genes present in all datasets were retained for downstream analysis. For each gene, we determined the number of datasets in which the correlation with *YBX1* met the following criteria: positive correlation (ρ ≥ 0.3 and *p* ≤ 0.01) or negative correlation (ρ ≤ −0.3 and *p* ≤ 0.01). Genes meeting these thresholds in ≥80% of datasets were considered consistently positively or negatively correlated with *YBX1*, respectively. For visualisation, correlation values for the filtered genes were arranged into a matrix with genes as rows and datasets as columns. Genes were ordered by mean correlation across datasets to highlight the most consistently associated genes. Side-bar annotations were added to indicate each gene’s mean correlation. Correlation values were visualised using colour gradients: negative correlations in blue, positive correlations from yellow to red, and values near zero in white. Heatmaps were generated with pheatmap, using hierarchical clustering for columns (datasets) and optional clustering for rows (genes), with row annotations for mean correlation values. To increase the robustness of results, genes with missing values in any dataset were removed. The analysis workflow produced summary statistics, including the number of genes consistently positively or negatively correlated with *YBX1* across datasets.

For proteomic analyses, Reverse Phase Protein Array (RPPA) data were obtained from TCGA datasets encompassing 31 tumour types. Spearman’s correlation analyses were performed to identify proteins significantly associated with YB-1 protein levels across each tumour type, using thresholds of ρ ≥ 0.3 or ρ ≤ −0.3 with q ≤ 0.05.

Pathway enrichment analysis for both transcriptomic and proteomic datasets was subsequently conducted using the Reactome Pathways 2024 database through EnrichR [62,63,64], with pathways of q-value ≤ 0.01 being considered significant.

Statistical analyses and graphical representations were conducted using GraphPad Prism (version 11.0.0), R (version 4.4.3), and Adobe Illustrator (version 29.5.1 (64 bit)).

### 4.4. Enrichment of Driver Mutations in High- or Low-YBX1 Tumours and the Pathway Analysis

To assess the relationship between *YBX1* mRNA expression and gene-specific mutation rates across multiple tumour types, we analysed mutation data from the 33 individual tumour types of the TCGA dataset. Tumours were stratified into high- and low-*YBX1* expression groups based on the 75th and 25th percentiles of *YBX1* mRNA expression within each tumour type, respectively. For each gene, the mutation rate was calculated in both groups, and a delta value (Δ = high − low) was computed to quantify enrichment of mutations in high-YBX1 tumours. Genes with missing mutation rates in either group were excluded from further analysis. The sample sizes for each cancer type are summarised in Table 3.

A curated list of cancer-associated driver genes was obtained from a previously published study by Bailey et al. [65], and the analysis was restricted to these genes. Each gene was classified as positive (Δ > 0), negative (Δ < 0), or unchanged (Δ = 0). For each gene, the number of tumour types with positive (pos) versus negative (neg) enrichment was summarised as a pos/neg ratio. Genes with a pos/neg ratio > 2 were considered consistently enriched in high-*YBX1* tumours, whereas genes with a ratio ≤ 0.8 were considered depleted.

Mutation enrichment status across tumour types was visualised using heatmaps. Genes were arranged in rows and tumour types in columns, with positive, negative, unchanged, or unknown status encoded as discrete categories. To facilitate visualisation, categorical status labels were mapped to numeric codes (positive = 1, negative = 2, unchanged = 3, unknown = 4) and heatmaps were generated using the pheatmap R package. Hierarchical clustering was applied to both genes and tumour types to reveal patterns of mutation enrichment across datasets. Separate heatmaps were produced for genes with pos/neg ratio > 2 and pos/neg ratio ≤ 0.8, highlighting consistent enrichment or depletion in high-*YBX1* tumours.

All analyses were performed using R (version 4.4.3) with the tidyverse, pheatmap, dplyr, tidyr, readr, and tibble packages. Processed data and code for generating mutation delta values and heatmaps are available upon request.

Gene ontology analysis was subsequently conducted using the GO Biological Processes 2025 database through EnrichR [62,63,64] with pathways of q-value < 0.01 being considered significant. These are summarised in Supplementary Table S3.

Statistical analyses and graphical representations were conducted using GraphPad Prism (version 11.0.0), R (version 4.4.3), and Adobe Illustrator (version 29.5.1 (64 bit)).

### 4.5. Multivariable Analysis of YBX1 mRNA Expression and Genomic Instability with Adjustment for Proliferative Activity

To assess whether the association between *YBX1* expression and genomic instability is independent of proliferative activity, we performed multivariable linear regression analyses adjusting for proliferation using both a single-gene marker and a multi-gene proliferation score. mRNA expression data for *YBX1*, *MKI67*, and genes included in the Hallmark G2M checkpoint gene set (MSigDB) were obtained from cBioPortal for the TCGA pan-cancer dataset (n = 9370). Corresponding data for the fraction of genome altered (FGA) and mutation count were also retrieved for the same samples.

Gene expression values were log2-transformed prior to analysis. Mutation count, defined as the total number of somatic mutations per sample, was log-transformed using log1p to account for skewness. FGA was used as a measure of chromosomal instability.

In addition to *MKI67*, a multi-gene proliferation score was derived using genes from the Hallmark G2M checkpoint gene set. For each gene, expression values were standardised across samples using z-score transformation, and a proliferation score for each sample was calculated as the mean of the standardised expression values across all genes in the set. To avoid redundancy, *MKI67* was excluded from the G2M gene set prior to score calculation.

Multivariable linear regression models were constructed to evaluate the association between *YBX1* expression and genomic instability metrics (FGA and mutation count), with adjustment for proliferation using either MKI67 expression or the G2M proliferation score. Separate models were fitted for each proliferation measure to avoid collinearity. Analyses were performed both at the pan-cancer level and within individual tumour types. For tumour-specific analyses, regression models were fitted separately for each cancer type to account for differences in baseline genomic features and biological context.

Model assumptions were assessed using standard diagnostic plots, and statistical significance was defined as a two-sided *p*-value < 0.05. Correlation between YBX1 expression and proliferation was assessed using Spearman correlation coefficients. Statistical analyses and graphical representations were conducted using R (version 4.4.3), and Adobe Illustrator (version 29.5.1 (64 bit)).

### 4.6. Molecular Characteristics Associated with YBX1 Levels

The molecular characteristics associated with *YBX1* expression levels were analysed using the BCGSC pan-cancer dataset, the TCGA pan-cancer dataset, and CPTAC studies across 10 out of 11 individual tumour types due to data availability (CPTAC_COAD, CPTAC_OV, CPTAC_HNSC, CPTAC_BRCA, CPTAC_LUSC, CPTAC_UCEC, CPTAC_CNS/Brain, CPTAC_RCC, CPTAC_PAAD, CPTAC_LUAD). The CPTAC studies were collectively assessed at the pan-cancer level and are hereafter referred to as the CPTAC pan-cancer dataset. Tumour samples in each pan-cancer dataset were stratified into high- and low-*YBX1* groups based on *YBX1* mRNA expression levels, with the high-*YBX1* group defined as the upper quartile (>75th percentile) and the low-*YBX1* group defined as the lower quartile (<25th percentile).

Molecular features analysed included mutation count, fraction of genome altered (FGA), homologous recombination deficiency (HRD) score, and hypoxia scores (Ragnum, Winter, Buffa). Mutation count, capturing missense, insertion, deletion, and frameshift mutations, was available across all datasets. FGA, reflecting the proportion of the genome affected by copy-number variations, was available only in TCGA, while HRD score, indicating homologous recombination repair defects, was available only in BCGSC. Together, these measures serve as surrogates for genomic stability. Comparisons between high- and low-*YBX1* tumours were performed using the Mann–Whitney U test, with *p* < 0.05 considered statistically significant.

Ragnum, Buffa, and Winter hypoxia scores are gene signature-based measures of transcriptional responses to low-oxygen levels within tumour microenvironments. This data was only available for the TCGA dataset. A Wilcoxon test was performed for comparisons between tumours with high- and low-*YBX1*, with a *p*-value < 0.05 considered to be statistically significant. The sample sizes for each molecular characteristic analysed are summarised in Table 4.

To assess whether these associations were also observed at the protein level, YB-1 protein abundance was analysed using Reverse Phase Protein Array (RPPA) data from TCGA tumour types. Tumours were stratified into high and low YB-1 protein expression groups based on the 75th and 25th percentiles of YB-1 protein levels, respectively. Molecular characteristics assessed at the protein level included mutation count, fraction of genome altered (FGA), and the Ragnum, Buffa, and Winter hypoxia scores.

A Wilcoxon test was performed for comparisons between tumours with high and low YBX1, with a *p*-value < 0.05 considered statistically significant. The sample sizes for each molecular characteristic analysed are summarised in Table 4.

Statistical analyses and graphical representations were conducted using GraphPad Prism (version 11.0.0) and Adobe Illustrator (version 29.5.1 (64 bit)).

### 4.7. Clinical Characteristics Associated with YBX1 mRNA Levels

Clinical characteristics associated with *YBX1* expression were analysed across the BCGSC and TCGA pan-cancer datasets by downloading the clinical data through cBioPortal. Tumour samples in each pan-cancer dataset were stratified into high- and low-*YBX1* groups based on *YBX1* mRNA expression levels, with the high-*YBX1* group defined as the upper quartile (>75th percentile) and the low-*YBX1* group defined as the lower quartile (<25th percentile).

The assessed parameters include the tumour stage derived from the American Joint Committee on Cancer (AJCC) tumour staging system, neoplasm histologic grade, and patient survival status.

Tumour stage (T1–4) represents the size and extent of invasion of the primary tumour. Neoplasm histologic grade describes the degree of abnormality (low-, mid-, and high-grade) of the cancerous tissue. Due to data availability, tumour stage and grade were analysed using the TCGA dataset only. A Chi-squared test was performed for comparisons between tumours with high- and low-*YBX1*, with a *p*-value < 0.05 considered to be statistically significant. The sample sizes for each of the clinal characteristics analysed are summarised in Table 4.

Patient survival was assessed using both progression-free survival (available for the TCGA study only) and overall survival (available for the TCGA and BCGSC studies). The sample sizes for each of the clinal characteristics analysed are summarised in Table 4. Progression-free survival refers to the length of time during and following treatment that a patient survives without the disease worsening, whereas overall survival refers to the length of time from diagnosis or treatment initiation that patients survive, regardless of disease status. A log-rank test was performed for comparisons between tumours with high and low *YBX1*, with a *p*-value < 0.05 considered to be statistically significant.

To assess whether these associations were also observed at the protein level, YB-1 protein abundance was analysed using Reverse Phase Protein Array (RPPA) data from the TCGA pan-cancer cohort. Tumours were stratified into high and low YB-1 protein expression groups based on the 75th and 25th percentiles of YB-1 protein levels, respectively. Associations between YB-1 protein expression and tumour stage, histologic grade, progression-free survival, and overall survival were assessed using the same statistical approaches as described for the mRNA-based analyses. Corresponding sample sizes for protein-level analyses are summarised in Table 4.

Statistical analyses and graphical representations were conducted using GraphPad Prism (version 11.0.0) and Adobe Illustrator (version 29.5.1 (64 bit)).

## 5. Conclusions

In summary, this study provides an integrated pan-cancer characterisation of tumour contexts associated with elevated YBX1 expression through the analysis of genomic, transcriptomic, proteomic, and clinical data across multiple independent cohorts. By assembling and systematically evaluating these data layers, we identify a reproducible pattern of tumour features that consistently co-occur with high YBX1 expression across diverse cancer types.

A key contribution of this work is the clarification of how YBX1 expression relates to genomic and clinical tumour characteristics at the pan-cancer level. In particular, our analyses delineate the extent to which associations between YBX1 expression and genomic instability-related features overlap with proliferative and mitotic-associated tumour biology, highlighting the importance of accounting for proliferative activity when interpreting these relationships.

Although the present study is inherently descriptive and based on bulk tumour data, it provides a consolidated framework that integrates prior observations with large-scale pan-cancer evidence. This framework helps to contextualise YBX1 expression within aggressive tumour phenotypes and establishes a foundation for future studies aimed at determining the context-specific functional roles of YB-1.

Future work will be required to determine the mechanistic basis and biological significance of these associations. Functional perturbation approaches, cell cycle-resolved analyses, and longitudinal models will be necessary to determine how *YBX1* relates to tumour evolution and therapeutic response in specific tumour contexts. Such studies will be critical for evaluating the utility of *YBX1* as a clinically informative biomarker and for defining conditions under which YB-1-associated pathways may represent actionable vulnerabilities.

## Figures and Tables

**Table 1:** Multivariable linear regression analysis of *YBX1* mRNA expression and genomic instability (FGA and mutation count), adjusted for proliferation using either MKI67 expression or a multi-gene cell cycle (G2M) score. β denotes the regression coefficient.

Model	Variable	FGA (β)	FGA (*p*-Value)	Mutation Count (β)	Mutation Count (*p*-Value)
*YBX1*	*YBX1*	0.05 ± 0.003	<2 × 10^−16^	0.58 ± 0.018	<2 × 10^−16^
					
*YBX1* + MKI67	*YBX1*	0.0138 ± 0.003	3.70 × 10^−5^	0.269 ± 0.018	<2 × 10^−16^
	MKI67	0.0281 ± 0.001	<2 × 10^−16^	0.245 ± 0.006	<2 × 10^−16^
					
*YBX1* + G2M Score	*YBX1*	0.0037 ± 0.003	0.258	0.427 ± 0.019	<2 × 10^−16^
	G2M Score	0.1496 ± 0.005	<2 × 10^−16^	0.515 ± 0.03	<2 × 10^−16^

**Table 2:** Summary of tumour types and sample sizes included in genomic and transcriptomic analyses of YBX1 and Spearman correlation analysis at the mRNA and protein level. N/A: not available.

Studies	Tumour Type	Genomic Profiles of *YBX1* (Sample Size)	Transcriptomic Profiles and Spearman’s Correlation of *YBX1* (Sample Size)	Proteomic Spearman’s Correlation of YB-1 (Sample Size)
BCGSC	Pan-cancer	570	570	-
TCGA	TCGA_UCS	56	56	47
	TCGA_DLBC	41	37	27
	TCGA_OV	511	201	304
	TCGA_UVM	80	80	12
	TCGA_SKCM	363	363	255
	TCGA_LAML	191	165	-
	TCGA_COADREAD *	532	524	417
	TCGA_COAD *	-	-	-
	TCGA_READ *	-	-	-
	TCGA_LUSC	484	82	309
	TCGA_MESO	86	119	59
	TCGA_THYM	123	484	90
	TCGA_CESC	287	275	152
	TCGA_TGCT	149	144	114
	TCGA_BLCA	407	402	338
	TCGA_SARC	253	251	217
	TCGA_HNSC	509	488	202
	TCGA_STAD	434	407	349
	TCGA_UCEC	511	507	406
	TCGA_ESCA	182	181	125
	TCGA_GBM	380	145	184
	TCGA_CHOL	36	36	30
	TCGA_PAAD	179	168	116
	TCGA_LUAD	511	503	356
	TCGA_LIHC	361	348	165
	TCGA_KIRC	400	352	315
	TCGA_KIRP	276	274	204
	TCGA_LGG	511	511	425
	TCGA_PRAD	489	488	346
	TCGA_THCA	487	480	358
	TCGA_BRCA	1053	994	810
	TCGA_ACC	89	76	44
	TCGA_KICH	65	65	62
	TCGA_PCPG	162	161	77
CPTAC	CPTAC_COAD	209	105	106
	CPTAC_OV	N/A	101	101
	CPTAC_HNSC	106	106	170
	CPTAC_BRCA	122	133	133
	CPTAC_LUSC	29	110	110
	CPTAC_UCEC	317	435	435
	CPTAC_CNS/Brain	95	221	221
	CPTAC_RCC	260	353	353
	CPTAC_PAAD	8	161	161
	CPTAC_LUAD	339	231	231
	CPTAC_GBM	192	N/A	N/A
Miscellaneous	IMvigor_mBLCA	347	347	347
	IMmotion_RCC	263	263	263
	DFCI_mM	122	122	122
	MSK_READ	725	100	100
	METABRIC_BRCA	1866	1866	1866
	GLASS_DG	329	355	355
	OHSU_LAML	562	451	451
	SU2C_mPRAD	429	208	208
	ASCproject_AS	274	157	157

* TCGA_COADREAD refers to colon adenocarcinoma and rectal adenocarcinoma combined; this was analysed separately as colon adenocarcinoma (TCGA_COAD) and rectal adenocarcinoma (TCGA_READ) in the Spearman correlation analysis at the transcriptomic level.

**Table 3:** Summary of tumour types and sample sizes included in the enrichment of driver mutations in high- or low-*YBX1* tumours.

Studies	Tumour Type	Enrichment of Driver Mutations	
		High-*YBX1* (Sample Size)	Low-*YBX1* (Sample Size)
TCGA (individual)	TCGA_UCS	14	14
	TCGA_DLBC	10	10
	TCGA_OV	50	50
	TCGA_UVM	20	20
	TCGA_SKCM	91	91
	TCGA_LAML	41	42
	TCGA_COADREAD	131	131
	TCGA_LUSC	21	21
	TCGA_MESO	30	30
	TCGA_THYM	117	117
	TCGA_CESC	69	69
	TCGA_TGCT	36	36
	TCGA_BLCA	101	101
	TCGA_SARC	63	63
	TCGA_HNSC	122	122
	TCGA_STAD	101	101
	TCGA_UCEC	127	127
	TCGA_ESCA	45	45
	TCGA_GBM	37	37
	TCGA_CHOL	9	9
	TCGA_PAAD	42	42
	TCGA_LUAD	126	126
	TCGA_LIHC	87	87
	TCGA_KIRC	88	88
	TCGA_KIRP	69	69
	TCGA_LGG	128	128
	TCGA_PRAD	122	122
	TCGA_THCA	120	120
	TCGA_BRCA	249	249
	TCGA_ACC	19	19
	TCGA_KICH	17	17
	TCGA_PCPG	41	41

**Table 4:** Summary of sample sizes included in the analyses of molecular and clinical characteristics associated with YBX1 mRNA and protein levels.

Studies			BCGSC	TCGA (mRNA)	TCGA (Protein)	CPTAC
Tumour Type			Pan-Cancer (Sample Size)	Pan-Cancer (Sample Size)	Pan-Cancer (Sample Size)	Pan-Cancer (Sample Size)
Molecular characteristics	Mutation counts	high-*YBX1*	142	2360	1689	483
		low-*YBX1*	143	2393	1689	278
	FGA	high-*YBX1*	N/A	2360	1689	N/A
		low-*YBX1*		2393	1689	
	HRD score	high-*YBX1*	144	N/A	N/A	N/A
		low-*YBX1*	143			
	Hypoxia scores *	high-*YBX1*	N/A	1722	1295	N/A
		low-*YBX1*		2171	1244	
Clinical characteristics	AJCC tumour stage	high-*YBX1*	N/A	1569	1392	N/A
		low-*YBX1*		1987	1149	
	Neoplasm histologic grade	high-*YBX1*	N/A	1027	556	N/A
		low-*YBX1*		684	774	
	Progression-free survival	high-*YBX1*	N/A	2399	N/A	N/A
		low-*YBX1*		2511		
	Overall survival	high-*YBX1*	143	2481	1671	N/A
		low-*YBX1*	144	2514	1678	

* Hypoxia scores: Ragnum, Wintere and Buffa.

## Data Availability

Data presented in the study are available in publicly accessible repositories, and the original data are openly available in cBioPortal [https://www.cbioportal.org/], accessed from 1 July 2025–20 April 2026).

## Supplementary Materials

The following supporting information can be downloaded at: https://www.mdpi.com/article/10.3390/ijms27104340/s1.
