# Supplementary material for: YBX1 Expression Marks Proliferative Tumour States with Context-Dependent Genomic Instability: A Pan-Cancer Analysis
Source: Int J Mol Sci. 2026 May 13;27(10):4340. doi: 10.3390/ijms27104340 (PMC13207732; doi:10.3390/ijms27104340)
Supplement: Supplementary file 1 [file ijms-27-04340-s001.zip › Figure S1_F.pdf]

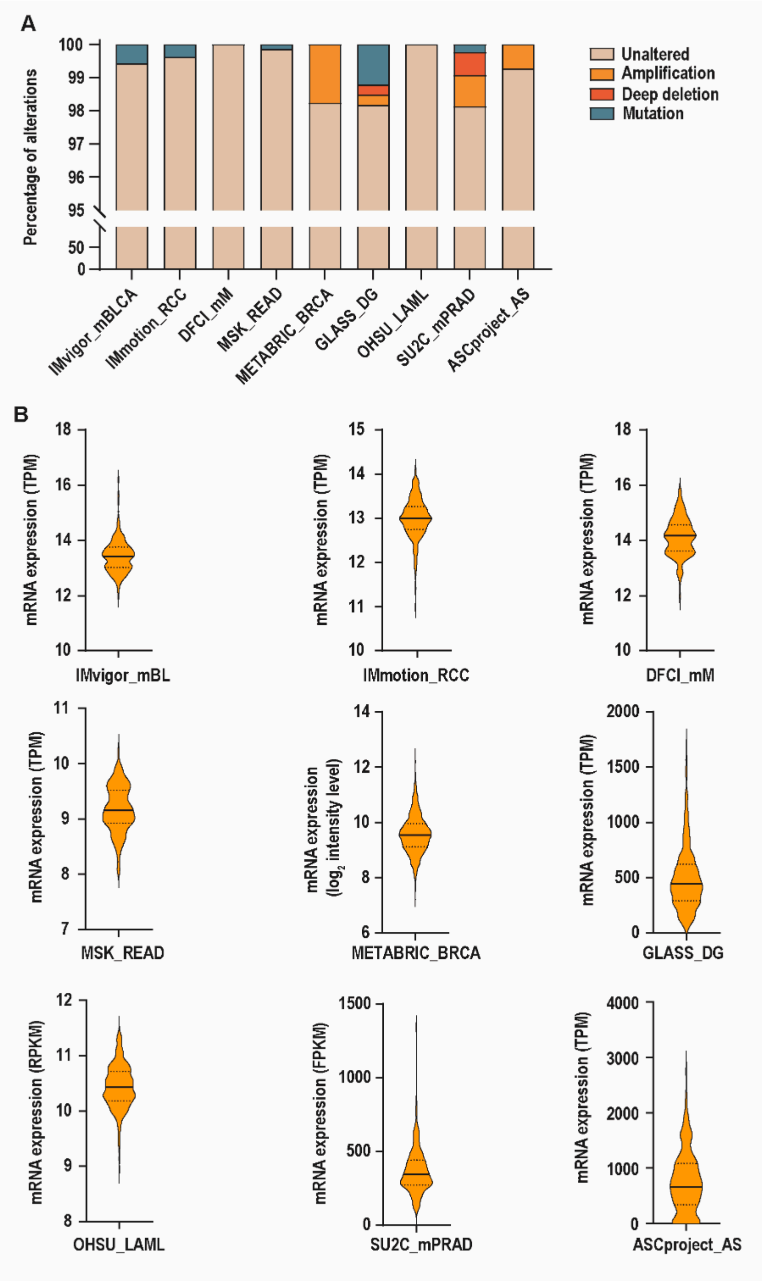

Figure S1. Genomic alterations and *YBX1* mRNA expression across additional independent datasets. A. Bar graphs showing the percentage of alterations in *YBX1* gene from tumour samples within datasets of IMvigor_mBLCAm, n = 347; IMmotion_RCC, n = 263; DFCI_mM, n = 122; MSK_READ, n = 725; METABRIC_BRCA, n = 1,866; GLASS_DG, n = 329; OHSU_LAML, n = 562; SU2C_mPRAD, n = 429; ASCproject_AS, n = 274. B. Violin plots showing the distribution of *YBX1* mRNA expression across each study. Each violin represents the density of expression values within a tumour group, with the width indicating frequency. The median expression is shown as a dark solid horizontal line, and interquartile range is indicated by dotted lines. IMvigor_mBLCAm, n = 347; IMmotion_RCC, n = 263; DFCI_mM, n = 122; MSK_READ, n = 100; METABRIC_BRCA, n = 1,866; GLASS_DG, n = 355; OHSU_LAML, n = 451; SU2C_mPRAD, n = 208; ASCproject_AS, n = 157.
