# Supplementary material for: YBX1 Expression Marks Proliferative Tumour States with Context-Dependent Genomic Instability: A Pan-Cancer Analysis
Source: Int J Mol Sci. 2026 May 13;27(10):4340. doi: 10.3390/ijms27104340 (PMC13207732; doi:10.3390/ijms27104340)
Supplement: Supplementary file 1 [file ijms-27-04340-s001.zip › Figure S2_F.pdf]

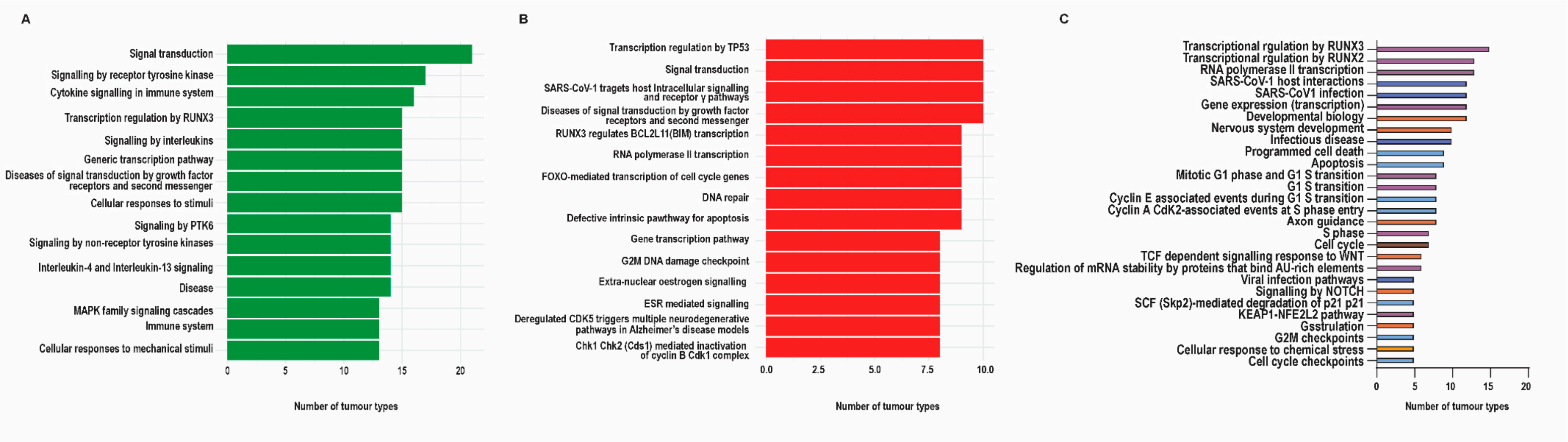

Figure S2: Top enriched pathways associated with YB-1 protein abundance and their overlap with pathways enriched in *YBX1* mRNA expression. (A–B) Bar graphs showing the number of tumour types in which the top 15 Reactome pathways (FDR ≤ 0.05) were enriched among proteins either **(A)** positively ($\rho \geq 0.3$, $q \leq 0.05$) or **(B)** negatively ($\rho \leq -0.3$, $q \leq 0.05$) associated with YB-1 protein abundance. **(C)** Bar graph showing the number of tumour types in which pathways were significantly enriched (FDR ≤ 0.05) for genes positively associated ($\rho \geq 0.3$, $q \leq 0.05$) with both *YBX1* mRNA expression and YB-1 protein abundance.
