# Supplementary material for: YBX1 Expression Marks Proliferative Tumour States with Context-Dependent Genomic Instability: A Pan-Cancer Analysis
Source: Int J Mol Sci. 2026 May 13;27(10):4340. doi: 10.3390/ijms27104340 (PMC13207732; doi:10.3390/ijms27104340)
Supplement: Supplementary file 1 [file ijms-27-04340-s001.zip › Figure S3_F.pdf]

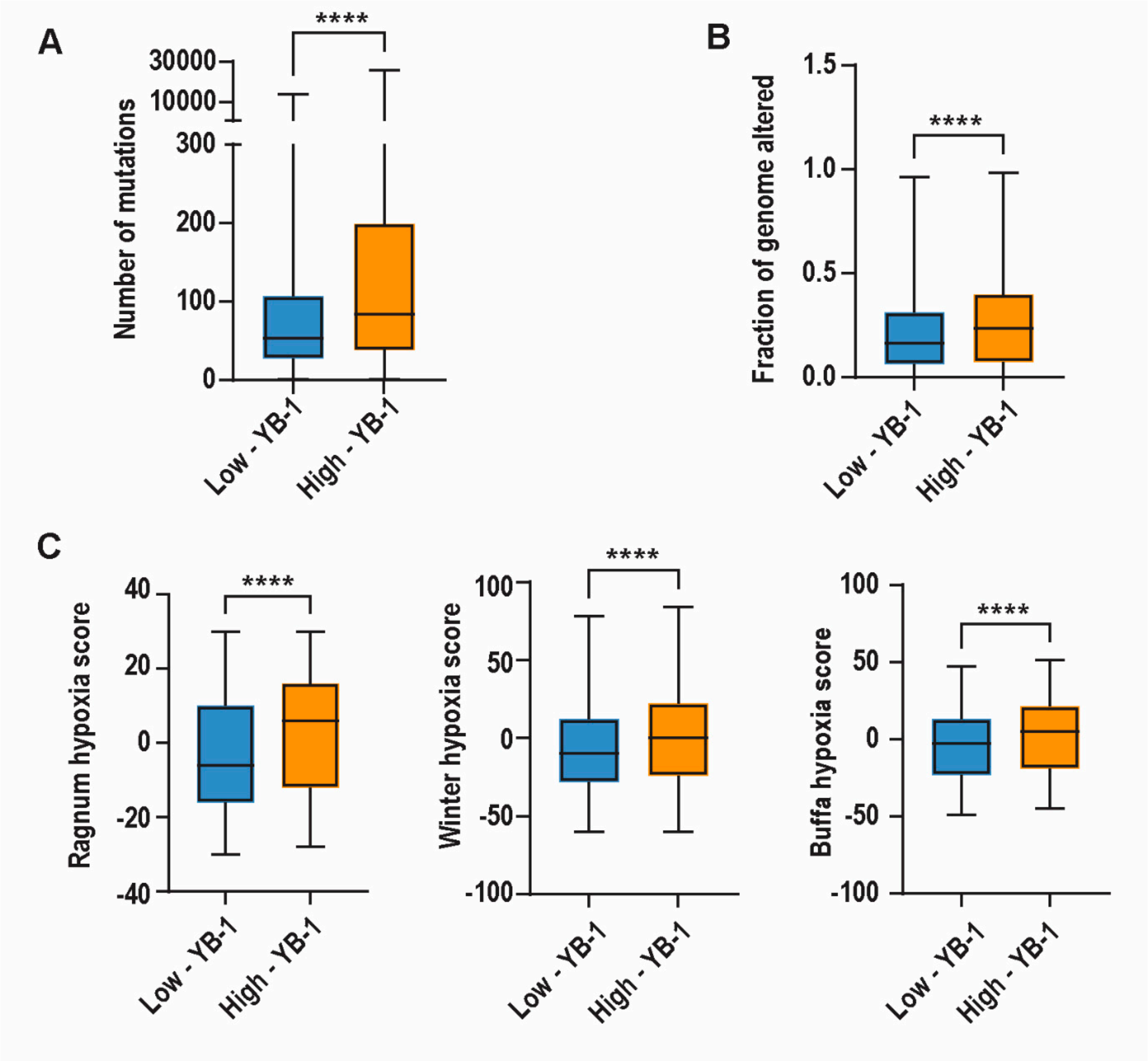

Figure S3. Tumours with high-YB-1 protein abundance display increased genomic instability and higher hypoxia score. A. Total mutation counts in high- and low-YB-1 tumours across the TCGA (high-YB-1, n = 1689; low-YB-1, n = 1689; ****p < 0.0001). Significance: Wilcoxon test, a p < 0.05 is considered statistically significant. **C.** Hypoxia scores in high-*YBX1* (n = 1722) and low-*YBX1* (n = 2171) tumours in the TCGA dataset (Ragnum: ****p < 0.0001; Buffa: ****p < 0.0001; and Winter: ****p < 0.0001). Significance: Wilcoxon test, a p < 0.05 is considered statistically significant. The central line represents the median, the box indicates the interquartile range (25th – 75th percentile), and the whiskers extend the most extreme data point within this range.
