# Supplementary material for: YBX1 Expression Marks Proliferative Tumour States with Context-Dependent Genomic Instability: A Pan-Cancer Analysis
Source: Int J Mol Sci. 2026 May 13;27(10):4340. doi: 10.3390/ijms27104340 (PMC13207732; doi:10.3390/ijms27104340)
Supplement: Supplementary file 1 [file ijms-27-04340-s001.zip › Figure S4_F.pdf]

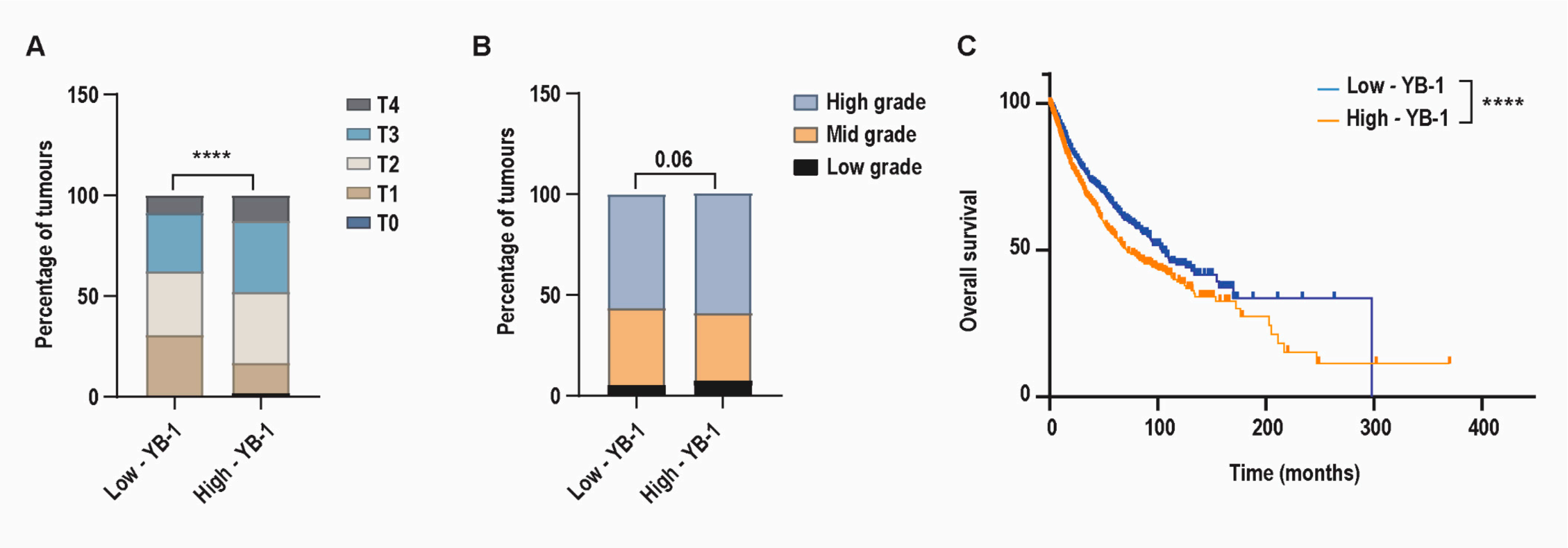

Figure S4: Tumours with high-YB-1 protein abundance exhibit advanced tumour stage and grade, with reduced survival. Bar graph comparing the percentage distribution of **A.** AJCC tumour stage in patients with high-YB-1 (n = 1392) and low-YB-1 (n = 1149) tumours across the TCGA dataset (****p < 0.0001). Significance: Chi-squared test, a p < 0.05 is considered statistically significant. T0/TIS: primary tumour not found or cannot be measured; T1-4: increasing in tumour size and invasion into nearby tissues. **B.** Neoplasm histologic grade in patients with high-YB-1 (n = 556) and low-YB-1 (n =774) tumours across the TCGA dataset (****p < 0.0001). Significance: Chi-squared test, p < 0.05 is considered statistically significant. Low grade: well-differentiated tumour cells; mid grade: moderately-differentiated tumour cells; high grade: poorly-differentiated to undifferentiated tumour cells. **C.** Overall survival of patients, TCGA dataset: high-YB-1 (n = 1671) and low-*YBX1* (n = 1678). **E.** Overall survival of patients, BCGSC dataset: high-*YBX1* (n = 143) and low-*YBX1* (n = 144). Significance: log-rank test, p < 0.05 is considered statistically significant, ****p < 0.0001.
