# Supplementary material for: YBX1 Expression Marks Proliferative Tumour States with Context-Dependent Genomic Instability: A Pan-Cancer Analysis
Source: Int J Mol Sci. 2026 May 13;27(10):4340. doi: 10.3390/ijms27104340 (PMC13207732; doi:10.3390/ijms27104340)
Supplement: Supplementary file 1 [file ijms-27-04340-s001.zip › Supplementary Table S1_V1_280426.pdf]

Table S1. List of Reactome pathways enriched for mRNAs significantly correlated with *YBX1* mRNA levels based on EnrichR analysis.

Term	P-value	Adjusted P-value
Metabolism of RNA	2.01E-10	5.17E-08
mRNA Splicing - Major Pathway	7.99E-05	1.28E-03
mRNA Splicing	9.79E-05	1.33E-03
Processing of Capped Intron-Containing Pre-mRNA	2.89E-04	3.39E-03
Metabolism of Polyamines	1.27E-03	6.33E-03
AUF1 (hnRNP D0) Binds and Destabilizes mRNA	1.06E-03	6.33E-03
Major Pathway of rRNA Processing in the Nucleolus and Cytosol	1.28E-03	6.33E-03
rRNA Processing in the Nucleus and Cytosol	1.48E-03	6.67E-03
rRNA Processing	2.44E-03	8.29E-03
Mitotic Metaphase and Anaphase	4.60E-06	3.96E-04
Mitotic Anaphase	4.50E-06	3.96E-04
M Phase	5.53E-05	1.10E-03
Separation of Sister Chromatids	4.60E-05	1.10E-03
Regulation of APC C Activators Between G1 S and Early Anaphase	6.34E-05	1.17E-03
Regulation of Mitotic Cell Cycle	8.50E-05	1.28E-03
Cell Cycle, Mitotic	2.57E-04	3.15E-03
FBXL7 Down-Regulates AURKA During Mitotic Entry and in Early Mitosis	1.06E-03	6.33E-03
Asymmetric Localization of PCP Proteins	1.56E-03	6.78E-03
The Role of GTSE1 in G2 M Progression After G2 Checkpoint	2.52E-03	8.33E-03
SCF-beta-TrCP Mediated Degradation of Emi1	1.48E-05	9.55E-04
Activation of APC C and APC C Cdc20 Mediated Degradation of Mitotic Proteins	5.29E-05	1.10E-03
APC C Cdc20 Mediated Degradation of Mitotic Proteins	5.04E-05	1.10E-03
APC C Cdh1 Mediated Degradation of Cdc20 and Other APC C Cdh1 Targets in Late Mitosis Early G1	4.58E-05	1.10E-03
APC Cdc20 Mediated Degradation of Cell Cycle Proteins Prior to Satisfation of the Checkpoint	4.58E-05	1.10E-03
Cdc20 Phospho-APC C Mediated Degradation of Cyclin A	4.36E-05	1.10E-03
APC C Cdc20 Mediated Degradation of Securin	3.35E-05	1.10E-03

**APC C-mediated Degradation of Cell Cycle Proteins**	8.50E-05	1.28E-03
**Ub-specific Processing Proteases**	1.30E-03	6.33E-03
**GLI3 Is Processed to GLI3R by the Proteasome**	1.33E-03	6.33E-03
**Degradation of GLI1 by the Proteasome**	1.33E-03	6.33E-03
**Degradation of GLI2 by the Proteasome**	1.33E-03	6.33E-03
**Degradation of DVL**	1.16E-03	6.33E-03
**GSK3B and BTRC CUL1-mediated-degradation of NFE2L2**	1.06E-03	6.33E-03
**Degradation of AXIN**	1.06E-03	6.33E-03
**Vif-mediated Degradation of APOBEC3G**	1.06E-03	6.33E-03
**Vpu Mediated Degradation of CD4**	9.61E-04	6.33E-03
**Autodegradation of the E3 Ubiquitin Ligase COP1**	9.13E-04	6.33E-03
**Ubiquitin Mediated Degradation of Phosphorylated Cdc25A**	9.13E-04	6.33E-03
**Ubiquitin-dependent Degradation of Cyclin D**	9.13E-04	6.33E-03
**Regulation of Activated PAK-2p34 by Proteasome Mediated Degradation**	8.22E-04	6.33E-03
**Regulation of Ornithine Decarboxylase (ODC)**	8.67E-04	6.33E-03
**SCF(Skp2)-mediated Degradation of P27 P21**	1.50E-03	6.67E-03
**Proteasome Assembly**	1.62E-03	6.78E-03
**Autodegradation of Cdh1 by Cdh1 APC C**	1.75E-03	6.82E-03
**Degradation of Beta-Catenin by the Destruction Complex**	3.08E-03	9.49E-03
**Regulation of Expression of SLITs and ROBOs**	3.42E-05	1.10E-03
**Signaling by ROBO Receptors**	8.94E-05	1.28E-03
**Axon Guidance**	3.11E-03	9.49E-03
**Cell Cycle Checkpoints**	2.06E-04	2.66E-03
**Stabilization of P53**	1.16E-03	6.33E-03
**Regulation of Apoptosis**	9.61E-04	6.33E-03
**p53-Independent DNA Damage Response**	9.13E-04	6.33E-03
**p53-Independent G1 S DNA Damage Checkpoint**	9.13E-04	6.33E-03
**p53-Dependent G1 DNA Damage Response**	1.68E-03	6.78E-03
**p53-Dependent G1 S DNA Damage Checkpoint**	1.68E-03	6.78E-03

**G1 S DNA Damage Checkpoints**	1.81E-03	6.87E-03
**Host Interactions of HIV Factors**	3.94E-04	4.42E-03
**Cross-presentation of Soluble Exogenous Antigens (Endosomes)**	8.22E-04	6.33E-03
**Viral Infection Pathways**	8.67E-04	6.33E-03
**HIV Infection**	2.16E-03	7.53E-03
**Infectious Disease**	3.01E-03	9.49E-03
**Cell Cycle**	7.33E-04	6.33E-03
**Orc1 Removal From Chromatin**	2.01E-03	7.41E-03
**CDK-mediated Phosphorylation and Removal of Cdc6**	2.15E-03	7.53E-03
**Cyclin E Associated Events During G1 S Transition**	3.16E-03	9.49E-03
**Cyclin A Cdk2-associated Events at S Phase Entry**	3.34E-03	9.89E-03
**NIK-->noncanonical NF-kB Signaling**	1.27E-03	6.33E-03
**Dectin-1 Mediated Noncanonical NF-kB Signaling**	1.44E-03	6.63E-03
**Activation of NF-kappaB in B Cells**	1.75E-03	6.82E-03
**Downstream Signaling Events of B Cell Receptor (BCR)**	2.75E-03	8.87E-03
**Hh Mutants Abrogate Ligand Secretion**	1.27E-03	6.33E-03
**Somitogenesis**	1.22E-03	6.33E-03
**Negative Regulation of NOTCH4 Signaling**	1.16E-03	6.33E-03
**Hh Mutants Are Degraded by ERAD**	1.11E-03	6.33E-03
**Hedgehog Ligand Biogenesis**	1.62E-03	6.78E-03
**Formation of Paraxial Mesoderm**	2.08E-03	7.45E-03
**Hedgehog 'On' State**	3.16E-03	9.49E-03
**Signaling by NOTCH4**	3.08E-03	9.49E-03
**Oxygen-dependent Proline Hydroxylation of Hypoxia-inducible Factor Alpha**	1.68E-03	6.78E-03
**Cellular Response to Hypoxia**	2.29E-03	7.89E-03
**Regulation of RUNX3 Expression and Activity**	1.22E-03	6.33E-03
**Defective CFTR Causes Cystic Fibrosis**	1.38E-03	6.48E-03
**Regulation of RAS by GAPs**	1.81E-03	6.87E-03
**Regulation of PTEN Stability and Activity**	1.88E-03	7.01E-03

**Regulation of RUNX2 Expression and Activity**	2.08E-03	7.45E-03
**ABC Transporter Disorders**	2.52E-03	8.33E-03
**RNA Polymerase II Transcription Termination**	2.67E-03	8.73E-03
