# Supplementary material for: YBX1 Expression Marks Proliferative Tumour States with Context-Dependent Genomic Instability: A Pan-Cancer Analysis
Source: Int J Mol Sci. 2026 May 13;27(10):4340. doi: 10.3390/ijms27104340 (PMC13207732; doi:10.3390/ijms27104340)
Supplement: Supplementary file 1 [file ijms-27-04340-s001.zip › Supplementary Table S2_F.pdf]

Table S2: Reactome pathways identified by EnrichR analysis of proteins significantly (FDR ≤ 0.05) correlated with YB-1 protein levels, along with the tumour types in which these pathways were commonly observed, comprising 283 positively and 163 negatively associated pathways.

Positively associated Reactome Enriched Pathways		
**Term**	**#Tumours**	**Tumour types**
**Signal Transduction**	21	BLCA, LGG, BRCA, COAD, DBCL, GBM, HNSC, KICH, KIRP, LIHC, LUAD, LUSC, OV, PAAD, PCPG, PRAD, SARC, SKCM, TGCT, THYM, THCA
**Signalling by Receptor Tyrosine Kinases**	17	BLCA, LGG, BRCA, COAD, DBCL, ESCA, HNSC, KICH, KIRP, LIHC, LUSC, OV, PCPG, PRAD, TGCT, THYM, THCA
**Cytokine Signalling in Immune System**	16	BLCA, LGG, COAD, DBCL, HNSC, KICH, KIRC, KIRP, LIHC, LUAD, LUSC, OV, PCPG, PRAD, SARC, THYM
**Cellular Responses to Stimuli**	15	BLCA, LGG, COAD, HNSC, KICH, KIRC, KIRP, LIHC, LUAD, LUSC, OV, PCPG, PRAD, SKCM, THYM
**Diseases of Signal Transduction by Growth Factor Receptors and Second Messengers**	15	LGG, COAD, DBCL, HNSC, KICH, KIRP, LIHC, LUSC, OV, PCPG, PRAD, SARC, TGCT, THYM, THCA
**Generic Transcription Pathway**	15	BLCA, COAD, HNSC, KICH, KIRP, LIHC, LUAD, LUSC, PCPG, PRAD, SARC, SKCM, TGCT, THCA, UCEC
**Signalling by Interleukins**	15	BLCA, COAD, DBCL, HNSC, KICH, KIRP, LIHC, LUAD, LUSC, MESO, OV, PRAD, SARC, THYM, UCEC
**Transcriptional Regulation by RUNX3**	15	BLCA, BRCA, COAD, HNSC, KICH, KIRP, LIHC, LUAD, LUSC, OV, PCPG, PRAD, SARC, TGCT, THCA
**Disease**	14	LGG, BRCA, COAD, DBCL, HNSC, KICH, KIRC, KIRP, LIHC, LUSC, OV, PCPG, PRAD, SARC
**Interleukin-4 and Interleukin-13 Signalling**	14	BLCA, COAD, DBCL, HNSC, KICH, KIRP, LIHC, LUAD, LUSC, MESO, OV, PRAD, SARC, THYM
**Signalling by Non-Receptor Tyrosine Kinases**	14	LGG, BRCA, COAD, GBM, HNSC, KIRP, LIHC, LUAD, LUSC, OV, PRAD, SARC, TGCT, THCA
**Signalling by PTK6**	14	LGG, BRCA, COAD, GBM, HNSC, KIRP, LIHC, LUAD, LUSC, OV, PRAD, SARC, TGCT, THCA
**Cellular Responses to Mechanical Stimuli**	13	BLCA, BRCA, COAD, DBCL, ESCA, HNSC, KICH, LIHC, LUAD, LUSC, PRAD, TGCT, THCA

**Immune System**	13	BRCA, COAD, DBCL, HNSC, KICH, KIRC, KIRP, LIHC, LUAD, LUSC, PCPG, PRAD, SARC
**MAPK Family Signalling Cascades**	13	BLCA, LGG, COAD, DBCL, HNSC, KICH, KIRP, LIHC, LUSC, OV, PCPG, PRAD, TGCT
**MAPK1 MAPK3 Signalling**	13	BLCA, LGG, COAD, DBCL, HNSC, KICH, KIRP, LIHC, LUSC, OV, PCPG, PRAD, TGCT
**PI3K AKT Signalling in Cancer**	13	LGG, COAD, GBM, HNSC, KICH, LIHC, LUSC, OV, PCPG, SARC, TGCT, THYM, THCA
**RAF MAP Kinase Cascade**	13	BLCA, LGG, COAD, DBCL, HNSC, KICH, KIRP, LIHC, LUSC, OV, PCPG, PRAD, TGCT
**RNA Polymerase II Transcription**	13	BLCA, COAD, HNSC, KICH, KIRP, LIHC, LUSC, PCPG, PRAD, SARC, SKCM, TGCT, UCEC
**Response of Endothelial Cells to Shear Stress**	13	BLCA, BRCA, COAD, DBCL, ESCA, HNSC, KICH, LIHC, LUAD, LUSC, PRAD, TGCT, THCA
**Transcriptional Regulation by RUNX2**	13	BLCA, COAD, HNSC, KICH, KIRP, LIHC, LUAD, LUSC, PRAD, SARC, SKCM, TGCT, THCA
**Developmental Biology**	12	COAD, GBM, HNSC, KICH, KIRP, LIHC, LUSC, OV, PAAD, PCPG, PRAD, TGCT
**Extra-nuclear Estrogen Signalling**	12	BLCA, COAD, ESCA, HNSC, KICH, KIRP, LIHC, LUAD, LUSC, PCPG, SARC, TGCT
**FOXO-mediated Transcription**	12	BLCA, COAD, ESCA, HNSC, KICH, KIRP, LIHC, LUSC, PCPG, PRAD, SARC, UCEC
**Gene Expression (Transcription)**	12	BLCA, COAD, HNSC, KICH, KIRP, LIHC, LUSC, PCPG, PRAD, SKCM, TGCT, UCEC
**Intrinsic Pathway for Apoptosis**	12	BLCA, LGG, COAD, DBCL, HNSC, KICH, KIRP, LIHC, LUSC, PCPG, PRAD, UCEC
**MTOR Signalling**	12	COAD, GBM, HNSC, KICH, KIRC, LIHC, LUAD, LUSC, PAAD, PCPG, PRAD, TGCT
**SARS-CoV-1 Infection**	12	BLCA, LGG, COAD, DBCL, ESCA, KICH, KIRP, LIHC, PCPG, PRAD, SARC, SKCM

**SARS-CoV-1-host Interactions**	12	BLCA, LGG, COAD, DBCL, ESCA, KICH, KIRP, LIHC, PCPG, PRAD, SARC, SKCM
**Negative Regulation of the PI3K AKT Network**	11	LGG, COAD, GBM, HNSC, KICH, KIRP, LIHC, LUSC, SARC, TGCT, THCA
**PI5P, PP2A and IER3 Regulate PI3K AKT Signalling**	11	LGG, COAD, GBM, HNSC, KICH, KIRP, LIHC, LUSC, SARC, TGCT, THCA
**PIP3 Activates AKT Signalling**	11	LGG, COAD, HNSC, KICH, KIRP, LIHC, LUSC, OV, PCPG, TGCT, THYM
**RUNX3 Regulates WNT Signalling**	11	BLCA, BRCA, COAD, HNSC, KIRP, LUAD, LUSC, OV, PRAD, SARC, TGCT
**SMAD2 SMAD3 SMAD4 Heterotrimer Regulates Transcription**	11	COAD, DBCL, HNSC, KICH, KIRP, LIHC, LUSC, OV, SARC, THCA, UCEC
**Signalling by ALK Fusions and Activated Point Mutants**	11	LGG, COAD, DBCL, HNSC, KICH, LIHC, LUAD, LUSC, OV, SARC, TGCT
**Signalling by ALK in Cancer**	11	LGG, COAD, DBCL, HNSC, KICH, LIHC, LUAD, LUSC, OV, SARC, TGCT
**Signalling by VEGF**	11	BRCA, COAD, ESCA, HNSC, KIRP, LIHC, LUSC, OV, PCPG, TGCT, THCA
**Transcriptional Regulation by TP53**	11	BLCA, COAD, HNSC, KICH, KIRP, LIHC, LUSC, PCPG, PRAD, SKCM, TGCT
**VEGFA-VEGFR2 Pathway**	11	BRCA, COAD, ESCA, HNSC, KIRP, LIHC, LUSC, OV, PCPG, TGCT, THCA
**BH3-only Proteins Associate With and Inactivate Anti-Apoptotic BCL-2 Members**	10	BLCA, COAD, DBCL, KICH, KIRP, LIHC, PRAD, SKCM, TGCT, THYM
**Cellular Responses to Stress**	10	LGG, COAD, HNSC, KICH, KIRC, KIRP, LIHC, LUSC, OV, SKCM
**Fibronectin Matrix Formation**	10	BLCA, DBCL, KICH, KIRC, LIHC, LUAD, LUSC, MESO, OV, TGCT
**Formation of Axial Mesoderm**	10	BLCA, BRCA, COAD, HNSC, KIRP, PCPG, PRAD, SARC, TGCT, UCEC
**Infectious Disease**	10	BRCA, COAD, DBCL, HNSC, KICH, KIRP, LIHC, LUSC, PRAD, SARC
**Intracellular Signalling by Second Messengers**	10	LGG, COAD, HNSC, KICH, KIRP, LIHC, LUSC, OV, PCPG, TGCT
**MAP2K and MAPK Activation**	10	DBCL, HNSC, KICH, KIRC, LIHC, LUAD, LUSC, OV, PCPG, PRAD
**Nervous System Development**	10	BLCA, COAD, GBM, HNSC, KICH, LIHC, PCPG, PRAD, TGCT, THCA
**Paradoxical Activation of RAF Signalling by Kinase Inactive BRAF**	10	DBCL, HNSC, KICH, KIRC, LIHC, LUAD, LUSC, OV, PCPG, PRAD
**Regulation of MITF-M-dependent Genes Involved in Cell Cycle and Proliferation**	10	BLCA, BRCA, COAD, HNSC, KIRP, LUAD, LUSC, OV, PRAD, SARC
**SARS-CoV-1 Targets Host Intracellular Signalling and Regulatory Pathways**	10	COAD, DBCL, ESCA, KICH, KIRP, LIHC, PCPG, PRAD, SARC, UCEC

**SARS-CoV-1-mediated Effects on Programmed Cell Death**	10	BLCA, LGG, COAD, DBCL, KICH, KIRP, LIHC, PRAD, SKCM, THYM
**STAT5 Activation Downstream of FLT3 ITD Mutants**	10	BLCA, COAD, DBCL, HNSC, KICH, KIRP, OV, PRAD, SKCM, THYM
**Signalling Downstream of RAS Mutants**	10	DBCL, HNSC, KICH, KIRC, LIHC, LUAD, LUSC, OV, PCPG, PRAD
**Signalling by FLT3 ITD and TKD Mutants**	10	BLCA, LGG, COAD, DBCL, HNSC, KICH, KIRP, LIHC, OV, THYM
**Signalling by High-Kinase Activity BRAF Mutants**	10	DBCL, HNSC, KICH, KIRC, LIHC, LUAD, LUSC, OV, PCPG, PRAD
**Signalling by Moderate Kinase Activity BRAF Mutants**	10	DBCL, HNSC, KICH, KIRC, LIHC, LUAD, LUSC, OV, PCPG, PRAD
**Signalling by RAF1 Mutants**	10	DBCL, HNSC, KICH, KIRC, LIHC, LUAD, LUSC, OV, PCPG, PRAD
**Signalling by RAS Mutants**	10	DBCL, HNSC, KICH, KIRC, LIHC, LUAD, LUSC, OV, PCPG, PRAD
**TP53 Regulates Metabolic Genes**	10	COAD, HNSC, KICH, KIRP, LIHC, LUAD, LUSC, PCPG, PRAD, TGCT
**Transcriptional Activity of SMAD2 SMAD3 SMAD4 Heterotrimer**	10	COAD, DBCL, HNSC, KICH, KIRP, LIHC, LUSC, SARC, THCA, UCEC
**Aberrant Regulation of Mitotic G1 S Transition in Cancer Due to RB1 Defects**	9	BLCA, KIRP, LIHC, LUAD, LUSC, OV, PCPG, PRAD, SARC
**Apoptosis**	9	BLCA, COAD, HNSC, KICH, KIRP, LIHC, LUSC, PCPG, PRAD
**Defective Binding of RB1 Mutants to E2F1,(E2F2, E2F3)**	9	BLCA, KIRP, LIHC, LUAD, LUSC, OV, PCPG, PRAD, SARC
**ERBB2 Activates PTK6 Signalling**	9	LGG, COAD, GBM, HNSC, KIRP, LUSC, OV, TGCT, THCA
**ERBB2 Regulates Cell Motility**	9	LGG, COAD, GBM, HNSC, KIRP, LUSC, OV, TGCT, THCA
**ESR-mediated Signalling**	9	BLCA, LGG, COAD, HNSC, KIRP, LIHC, LUSC, PCPG, UCEC
**FOXO-mediated Transcription of Cell Cycle Genes**	9	BLCA, COAD, ESCA, KICH, KIRP, OV, PCPG, SARC, UCEC
**Frs2-mediated Activation**	9	COAD, DBCL, HNSC, KICH, KIRP, LIHC, LUSC, PCPG, PRAD
**GRB7 Events in ERBB2 Signalling**	9	LGG, COAD, GBM, HNSC, LUSC, OV, PRAD, TGCT, THCA
**Integrin Cell Surface Interactions**	9	BLCA, COAD, DBCL, ESCA, KICH, LUAD, OV, TGCT, THCA
**MITF-M-regulated Melanocyte Development**	9	COAD, HNSC, KICH, KIRP, LIHC, LUAD, LUSC, PRAD, SARC
**Negative Feedback Regulation of MAPK Pathway**	9	COAD, DBCL, HNSC, KICH, KIRP, LIHC, LUSC, PCPG, PRAD
**PI3K Events in ERBB2 Signalling**	9	LGG, COAD, GBM, HNSC, KIRP, LUSC, OV, TGCT, THCA
**Programmed Cell Death**	9	BLCA, COAD, HNSC, KICH, KIRP, LIHC, LUSC, PCPG, PRAD

**Prolonged ERK Activation Events**	9	COAD, DBCL, HNSC, KICH, KIRP, LIHC, LUSC, PCPG, PRAD
**RAF-independent MAPK1 3 Activation**	9	BLCA, COAD, DBCL, HNSC, KICH, KIRP, LIHC, LUSC, PCPG
**RUNX3 Regulates CDKN1A Transcription**	9	KICH, KIRP, LIHC, MESO, OV, PCPG, SARC, SKCM, UCEC
**SARS-CoV-2 Targets Host Intracellular Signalling and Regulatory Pathways**	9	BLCA, COAD, ESCA, HNSC, KICH, KIRP, LUSC, PCPG, PRAD
**Signalling by BRAF and RAF1 Fusions**	9	DBCL, HNSC, KICH, KIRC, LIHC, LUAD, LUSC, PCPG, PRAD
**Signalling by Nuclear Receptors**	9	BLCA, LGG, COAD, HNSC, KIRP, LIHC, LUSC, TGCT, UCEC
**Signalling by TGFB Family Members**	9	BLCA, COAD, HNSC, KICH, KIRP, LIHC, LUSC, SARC, SKCM
**Apoptotic Factor-Mediated Response**	8	COAD, HNSC, KICH, KIRP, LIHC, LUSC, PCPG, UCEC
**Axon Guidance**	8	COAD, GBM, HNSC, KICH, LIHC, PAAD, PCPG, TGCT
**Binding of TCF LEF CTNNB1 to Target Gene Promoters**	8	BRCA, COAD, HNSC, KIRP, LUSC, OV, PRAD, TGCT
**Constitutive Signalling by Aberrant PI3K in Cancer**	8	LGG, COAD, GBM, HNSC, KICH, SARC, TGCT, THCA
**Cyclin A Cdk2-associated Events at S Phase Entry**	8	COAD, HNSC, LIHC, LUAD, LUSC, OV, PRAD, SARC
**Cyclin E Associated Events During G1 S Transition**	8	COAD, HNSC, LIHC, LUAD, LUSC, OV, PRAD, SARC
**Downregulation of ERBB2 Signalling**	8	LGG, COAD, GBM, HNSC, LUSC, OV, TGCT, THCA
**Downregulation of SMAD2 3 SMAD4 Transcriptional Activity**	8	DBCL, KICH, KIRP, LIHC, LUSC, SARC, THCA, UCEC
**ECM Proteoglycans**	8	BLCA, DBCL, KICH, KIRC, LIHC, LUAD, OV, TGCT
**Oestrogen-dependent Nuclear Events Downstream of ESR-membrane Signalling**	8	COAD, HNSC, KIRP, LIHC, LUAD, LUSC, PCPG, SARC
**FLT3 Signalling in Disease**	8	LGG, COAD, DBCL, HNSC, KICH, LIHC, OV, THYM
**G1 S Transition**	8	BLCA, COAD, HNSC, LUAD, LUSC, OV, PRAD, SARC
**GRB2 Events in ERBB2 Signalling**	8	LGG, COAD, GBM, HNSC, KIRP, LUSC, TGCT, THCA
**MITF-M-dependent Gene Expression**	8	BRCA, COAD, HNSC, KIRP, LUAD, LUSC, PRAD, SARC
**Mitotic G1 Phase and G1 S Transition**	8	BLCA, COAD, HNSC, LUAD, LUSC, OV, PCPG, SARC
**Oncogenic MAPK Signalling**	8	DBCL, HNSC, KICH, LIHC, LUAD, LUSC, PCPG, PRAD
**PTK6 Regulates Cell Cycle**	8	BLCA, KIRP, LIHC, LUAD, LUSC, PCPG, PRAD, SARC
**RUNX2 Regulates Bone Development**	8	BLCA, KICH, LIHC, LUSC, PAAD, SKCM, TGCT, THCA

**RUNX3 Regulates BCL2L11 (BIM) Transcription**	8	KICH, KIRP, LIHC, MESO, PCPG, SARC, SKCM, UCEC
**Rab Regulation of Trafficking**	8	LGG, COAD, HNSC, LIHC, LUSC, PCPG, PRAD, SKCM
**SARS-CoV Infections**	8	COAD, DBCL, HNSC, KICH, KIRP, LIHC, LUSC, PCPG
**SHC1 Events in ERBB2 Signalling**	8	LGG, COAD, GBM, HNSC, LUSC, OV, TGCT, THCA
**Signalling by Activin**	8	COAD, HNSC, KIRP, LIHC, LUSC, PCPG, SARC, UCEC
**Signalling by ERBB2**	8	LGG, COAD, GBM, HNSC, LUSC, OV, TGCT, THCA
**Signalling by ERBB2 TMD JMD Mutants**	8	LGG, COAD, GBM, HNSC, LUSC, OV, TGCT, THCA
**Signalling by NODAL**	8	COAD, HNSC, KIRP, LIHC, LUSC, PCPG, SARC, UCEC
**Signalling by TGF-beta Receptor Complex**	8	COAD, DBCL, HNSC, KIRP, LIHC, LUSC, SARC, THCA
**Turbulent Flow Shear Stress Activates Signalling by PIEZO1 and Integrins in Endothelial Cells**	8	BLCA, DBCL, KICH, KIRC, LUAD, LUSC, OV, TGCT
**mTORC1-mediated Signalling**	8	GBM, HNSC, KICH, KIRC, LIHC, LUAD, PAAD, PCPG
**AKT Phosphorylates Targets in the Cytosol**	7	COAD, HNSC, KICH, LIHC, LUSC, OV, PCPG
**Activation of BAD and Translocation to Mitochondria**	7	BLCA, COAD, HNSC, KICH, LUSC, PCPG, PRAD
**Cell Cycle**	7	COAD, HNSC, KICH, KIRP, LUSC, OV, PRAD
**Cellular Senescence**	7	COAD, HNSC, KIRP, LIHC, LUSC, OV, SKCM
**Constitutive Signalling by AKT1 E17K in Cancer**	7	COAD, HNSC, KICH, LIHC, LUSC, OV, PCPG
**DNA Double-Strand Break Repair**	7	COAD, HNSC, KICH, KIRP, PAAD, PRAD, SKCM
**Downregulation of ERBB2 ERBB3 Signalling**	7	LGG, COAD, GBM, HNSC, LUSC, OV, THCA
**Drug-mediated Inhibition of CDK4 CDK6 Activity**	7	BLCA, KIRP, LIHC, LUAD, LUSC, PRAD, SARC
**Oestrogen-stimulated Signalling Through PRKCZ**	7	LGG, COAD, HNSC, KICH, KIRP, LIHC, LUSC
**Formation of Definitive Endoderm**	7	BRCA, COAD, HNSC, KIRP, PCPG, SARC, UCEC
**GAB1 Signalosome**	7	BRCA, COAD, HNSC, KIRP, LIHC, OV, PCPG
**GRB2 SOS Provides Linkage to MAPK Signalling for Integrins**	7	BLCA, DBCL, KIRC, LUAD, LUSC, OV, PCPG
**Germ Layer Formation at Gastrulation**	7	BRCA, COAD, HNSC, KIRP, PCPG, SARC, UCEC
**IFNG Signalling Activates MAPKs**	7	COAD, HNSC, KICH, KIRP, LIHC, LUSC, PRAD

**InlA-mediated Entry of Listeria Monocytogenes Into Host Cells**	7	BRCA, COAD, HNSC, KIRP, PCPG, PRAD, TGCT
**Insulin Receptor Signalling Cascade**	7	LGG, COAD, HNSC, KICH, LIHC, LUSC, SARC
**Loss of Function of SMAD2 3 in Cancer**	7	KIRP, LIHC, MESO, PCPG, SARC, SKCM, UCEC
**Loss of Function of TGFBR1 in Cancer**	7	KIRP, LIHC, MESO, PCPG, SARC, SKCM, UCEC
**NFE2L2 Regulating Tumorigenic Genes**	7	BLCA, COAD, DBCL, KIRP, PRAD, SKCM, THYM
**RUNX2 Regulates Osteoblast Differentiation**	7	BLCA, HNSC, KICH, LUSC, PCPG, TGCT, THCA
**Regulation of PTEN Gene Transcription**	7	HNSC, KICH, KIRC, KIRP, LIHC, LUAD, LUSC
**S Phase**	7	COAD, HNSC, KICH, LIHC, LUSC, OV, SARC
**SMAD2 3 Phosphorylation Motif Mutants in Cancer**	7	KIRP, LIHC, MESO, PCPG, SARC, SKCM, UCEC
**Sensing of DNA Double Strand Breaks**	7	LGG, COAD, HNSC, KIRP, LUSC, PRAD, SKCM
**Signalling by ERBB2 ECD Mutants**	7	COAD, GBM, HNSC, KIRP, LUSC, TGCT, THCA
**Signalling by ERBB2 KD Mutants**	7	LGG, GBM, HNSC, LUSC, OV, TGCT, THCA
**Signalling by ERBB2 in Cancer**	7	LGG, GBM, HNSC, LUSC, OV, TGCT, THCA
**Signalling by Insulin Receptor**	7	LGG, COAD, HNSC, KICH, LIHC, LUSC, SARC
**Signalling to ERKs**	7	DBCL, HNSC, KICH, LIHC, LUSC, PCPG, PRAD
**Spry Regulation of FGF Signalling**	7	COAD, HNSC, KICH, KIRP, LIHC, LUSC, PCPG
**TFAP2 (AP-2) Family Regulates Transcription of Growth Factors and Their Receptors**	7	COAD, GBM, HNSC, KIRP, LUSC, TGCT, THCA
**TGFBR1 KD Mutants in Cancer**	7	KIRP, LIHC, MESO, PCPG, SARC, SKCM, UCEC
**Transcriptional Regulation by the AP-2 (TFAP2) Family of Transcription Factors**	7	COAD, GBM, HNSC, LUSC, OV, TGCT, THCA
**Transcriptional and Post-Translational Regulation of MITF-M Expression and Activity**	7	BRCA, COAD, HNSC, KICH, KIRP, LIHC, LUSC
**Uptake and Function of Diphtheria Toxin**	7	BRCA, COAD, KICH, KIRP, LIHC, MESO, SKCM
**VEGFR2 Mediated Vascular Permeability**	7	BRCA, COAD, ESCA, HNSC, KIRP, LUSC, TGCT
**p130Cas Linkage to MAPK Signalling for Integrins**	7	BLCA, DBCL, KIRC, LUAD, LUSC, OV, PCPG
**ALK Mutants Bind TKIs**	6	BLCA, DBCL, KIRC, LUAD, LUSC, OV

**Aberrant Regulation of Mitotic Cell Cycle Due to RB1 Defects**	6	LIHC, LUAD, LUSC, OV, PRAD, SARC
**Activated NTRK3 Signals Through PI3K**	6	LGG, HNSC, LIHC, PCPG, SARC, TGCT
**Activation of BH3-only Proteins**	6	COAD, HNSC, KICH, LIHC, LUSC, PRAD
**Bacterial Infection Pathways**	6	BRCA, COAD, HNSC, KICH, KIRP, LIHC
**CDH11 Homotypic and Heterotypic Interactions**	6	BRCA, COAD, HNSC, KIRP, PRAD, TGCT
**Cell Junction Organization**	6	BRCA, COAD, HNSC, KIRP, LIHC, PRAD
**Cyclin D Associated Events in G1**	6	LIHC, LUAD, LUSC, PCPG, PRAD, SARC
**Cytochrome C-Mediated Apoptotic Response**	6	COAD, HNSC, KICH, KIRP, LIHC, LUSC
**Deadenylation of mRNA**	6	GBM, HNSC, KICH, LUAD, PAAD, PCPG
**Disassembly of the Destruction Complex and Recruitment of AXIN to the Membrane**	6	BRCA, COAD, ESCA, KICH, KIRP, TGCT
**Diseases of Mitotic Cell Cycle**	6	LIHC, LUAD, LUSC, OV, PRAD, SARC
**Energy Dependent Regulation of mTOR by LKB1-AMPK**	6	HNSC, KIRC, LIHC, LUAD, PCPG, TGCT
**Formation of Apoptosome**	6	COAD, HNSC, KICH, KIRP, LIHC, LUSC
**Formation of the Nephric Duct**	6	BRCA, COAD, HNSC, KIRP, THYM, UCEC
**G1 Phase**	6	LIHC, LUAD, LUSC, PCPG, PRAD, SARC
**G2 M DNA Damage Checkpoint**	6	LGG, HNSC, KICH, KIRP, PRAD, SKCM
**Gain-of-function MRAS Complexes Activate RAF Signalling**	6	HNSC, KICH, LIHC, LUSC, PCPG, PRAD
**HDR Through MMEJ (alt-NHEJ)**	6	COAD, HNSC, KIRP, LUSC, PRAD, SKCM
**HDR Through Single Strand Annealing (SSA)**	6	COAD, HNSC, LUSC, PAAD, PRAD, SKCM
**High Laminar Flow Shear Stress Activates Signalling by PIEZO1 and PECAM1 CDH5 KDR in Endothelial Cell**	6	BRCA, COAD, ESCA, HNSC, LIHC, THCA
**IGF1R Signalling Cascade**	6	LGG, COAD, HNSC, LIHC, LUSC, SARC
**IRS-mediated Signalling**	6	LGG, COAD, HNSC, LIHC, LUSC, SARC
**IRS-related Events Triggered by IGF1R**	6	LGG, COAD, HNSC, LIHC, LUSC, SARC

**Innate Immune System**	6	BRCA, COAD, HNSC, LIHC, LUSC, PRAD
**Integrin Signalling**	6	DBCL, HNSC, KIRC, LUAD, LUSC, OV
**LRR FLII-interacting Protein 1 (LRRFIP1) Activates Type I IFN Production**	6	BRCA, COAD, HNSC, KIRP, PRAD, TGCT
**MET Activates PTK2 Signalling**	6	DBCL, KIRC, LUAD, LUSC, OV, TGCT
**MET Promotes Cell Motility**	6	DBCL, KIRC, LUAD, LUSC, OV, TGCT
**Metabolism of Nitric Oxide NOS3 Activation and Regulation**	6	BLCA, COAD, ESCA, HNSC, KIRP, LUSC
**NOSTRIN Mediated eNOS Trafficking**	6	BLCA, COAD, ESCA, KICH, KIRP, TGCT
**Negative Regulation of MAPK Pathway**	6	DBCL, HNSC, KICH, LIHC, LUSC, PRAD
**Non-integrin membrane-ECM Interactions**	6	DBCL, KIRC, LUAD, LUSC, OV, TGCT
**Nuclear Events Stimulated by ALK Signalling in Cancer**	6	COAD, HNSC, KICH, LIHC, LUSC, PAAD
**Oncogene Induced Senescence**	6	COAD, KIRP, LIHC, LUSC, OV, UCEC
**Platelet Activation, Signalling and Aggregation**	6	DBCL, HNSC, KICH, LIHC, LUSC, OV
**Post-translational Protein Modification**	6	LGG, KICH, OV, PAAD, SKCM, UCEC
**RAF Activation**	6	DBCL, KICH, LIHC, LUSC, PCPG, PRAD
**RHO GTPases Activate PAKs**	6	BRCA, COAD, GBM, LUSC, PAAD, PRAD
**RSK Activation**	6	COAD, HNSC, KICH, KIRP, LIHC, LUSC
**RUNX3 Regulates p14-ARF**	6	BLCA, KIRP, LUAD, LUSC, PRAD, SARC
**Regulation of CDH19 Expression and Function**	6	BRCA, COAD, HNSC, KIRP, PRAD, TGCT
**Regulation of Cytoskeletal Remodelling and Cell Spreading by IPP Complex Components**	6	BRCA, COAD, HNSC, KIRP, OV, SKCM
**Regulation of TP53 Activity**	6	BLCA, COAD, HNSC, LUSC, PRAD, SKCM
**Regulation of mRNA Stability by Proteins That Bind AU-rich Elements**	6	GBM, HNSC, KICH, LUAD, PCPG, PRAD
**Regulation of the Apoptosome Activity**	6	COAD, HNSC, KICH, KIRP, LIHC, LUSC
**Ribosomal Scanning and Start Codon Recognition**	6	GBM, KICH, LIHC, LUAD, PAAD, PCPG

**SHOC2 M1731 Mutant Abolishes MRAS Complex Function**	6	HNSC, KICH, LIHC, LUSC, PCPG, PRAD
**Signal Attenuation**	6	COAD, HNSC, KIRP, LUSC, SARC, TGCT
**Signal Transduction by L1**	6	COAD, DBCL, HNSC, KIRP, LIHC, LUSC
**Signalling by ERBB4**	6	BLCA, LGG, HNSC, PRAD, TGCT, UCEC
**Signalling by LTK in Cancer**	6	COAD, HNSC, KICH, KIRP, LIHC, LUSC
**Signalling by MAPK Mutants**	6	COAD, HNSC, KICH, KIRP, LIHC, LUSC
**Signalling by MRAS-complex Mutants**	6	HNSC, KICH, LIHC, LUSC, PCPG, PRAD
**Signalling by NTRKs**	6	HNSC, KICH, LIHC, PCPG, PRAD, SARC
**Signalling by TGF-beta Receptor Complex in Cancer**	6	KIRP, MESO, PCPG, SARC, SKCM, UCEC
**Signalling by Type 1 Insulin-like Growth Factor 1 Receptor (IGF1R)**	6	LGG, COAD, HNSC, LIHC, LUSC, SARC
**Suppression of Apoptosis**	6	COAD, HNSC, KICH, KIRP, LIHC, LUSC
**Syndecan Interactions**	6	DBCL, KIRC, LUAD, LUSC, OV, TGCT
**TBC RABGAPs**	6	LGG, HNSC, LIHC, PCPG, PRAD, SKCM
**TCF Dependent Signalling in Response to WNT**	6	BRCA, COAD, HNSC, KICH, KIRP, LUSC
**Transcriptional Regulation by VENTX**	6	BRCA, KIRP, LUAD, LUSC, PRAD, SARC
**Translation Initiation Complex Formation**	6	GBM, KICH, LIHC, LUAD, PAAD, PCPG
**Z-decay Degradation of Maternal mRNAs by Zygotically Expressed Factors**	6	GBM, HNSC, KICH, LUAD, PAAD, PCPG
**eNOS Activation**	6	BLCA, COAD, ESCA, HNSC, KIRP, LUSC
**mRNA Activation Upon Binding of the Cap-Binding Complex and eIFs, and Subsequent Binding to 43S**	6	GBM, KICH, LIHC, LUAD, PAAD, PCPG
**ARMS-mediated Activation**	5	HNSC, KICH, LIHC, PCPG, PRAD
**Apoptotic Cleavage of Cell Adhesion Proteins**	5	BRCA, COAD, HNSC, KIRP, PRAD
**CD28 Co-Stimulation**	5	COAD, HNSC, LIHC, LUSC, PCPG
**CD28 Dependent PI3K Akt Signalling**	5	COAD, HNSC, LIHC, LUSC, PCPG
**Ca2+ Pathway**	5	BRCA, COAD, HNSC, LUSC, OV
**Cap-dependent Translation Initiation**	5	GBM, KICH, LUAD, PAAD, PCPG

**Cell Cycle Checkpoints**	5	LGG, HNSC, KICH, PRAD, SKCM
**Cell-Cell Communication**	5	BRCA, COAD, HNSC, KIRP, LIHC
**Cell-extracellular Matrix Interactions**	5	BRCA, COAD, HNSC, KIRP, OV
**Cellular Response to Chemical Stress**	5	COAD, HNSC, KICH, LUSC, OV
**Cellular Response to Heat Stress**	5	HNSC, KICH, KIRP, LIHC, LUAD
**Chk1 Chk2(Cds1) Mediated Inactivation of Cyclin B Cdk1 Complex**	5	BLCA, KICH, PCPG, PRAD, SKCM
**Constitutive Signalling by Overexpressed ERBB2**	5	COAD, GBM, HNSC, LUSC, THCA
**Costimulation by the CD28 Family**	5	COAD, HNSC, LIHC, LUSC, PCPG
**DNA Damage Telomere Stress Induced Senescence**	5	LGG, HNSC, LUSC, PRAD, SKCM
**DNA Repair**	5	HNSC, KIRP, PAAD, PRAD, SKCM
**Deactivation of the Beta-Catenin Transactivating Complex**	5	BRCA, COAD, HNSC, LUSC, PRAD
**Elastic Fibre Formation**	5	DBCL, KIRC, LUAD, LUSC, OV
**Oestrogen-dependent Gene Expression**	5	BLCA, LUAD, LUSC, SARC, UCEC
**Eukaryotic Translation Initiation**	5	GBM, KICH, LUAD, PAAD, PCPG
**FLT3 Signalling**	5	COAD, HNSC, KICH, LIHC, LUSC
**Fcgamma Receptor (FCGR) Dependent Phagocytosis**	5	COAD, GBM, HNSC, LIHC, LUSC
**G2 M Checkpoints**	5	HNSC, KICH, KIRP, PRAD, SKCM
**GP1b-IX-V Activation Signalling**	5	KICH, LIHC, LUSC, PCPG, PRAD
**GPER1 Signalling**	5	DBCL, KIRC, LUAD, LUSC, OV
**GTP Hydrolysis and Joining of the 60S Ribosomal Subunit**	5	GBM, KICH, LUAD, PAAD, PCPG
**Gastrin-CREB Signalling Pathway via PKC and MAPK**	5	COAD, HNSC, KIRP, LUSC, OV
**Gastrulation**	5	BRCA, KIRP, PRAD, SARC, TGCT
**Golgi Cisternae Pericentriolar Stack Reorganization**	5	BLCA, COAD, HNSC, KIRP, LUSC
**HDR Through Homologous Recombination (HRR) or Single Strand Annealing (SSA)**	5	COAD, HNSC, KIRP, PRAD, SKCM

**Homology Directed Repair**	5	COAD, HNSC, KIRP, PRAD, SKCM
**ISG15 Antiviral Mechanism**	5	GBM, KICH, KIRC, LUAD, PCPG
**KEAP1-NFE2L2 Pathway**	5	COAD, HNSC, KICH, LUSC, OV
**L13a-mediated Translational Silencing of Ceruloplasmin Expression**	5	GBM, KICH, LUAD, PAAD, PCPG
**M-decay Degradation of Maternal mRNAs by Maternally Stored Factors**	5	GBM, KICH, LUAD, PAAD, PCPG
**MAP Kinase Activation**	5	COAD, DBCL, HNSC, LIHC, LUSC
**Molecules Associated With Elastic Fibres**	5	DBCL, KIRC, LUAD, LUSC, OV
**Negative Regulation of FGFR1 Signalling**	5	HNSC, KICH, LIHC, LUSC, PCPG
**Negative Regulation of FGFR2 Signalling**	5	HNSC, KICH, LIHC, LUSC, PCPG
**Negative Regulation of FGFR3 Signalling**	5	HNSC, KICH, LIHC, LUSC, PCPG
**Negative Regulation of FGFR4 Signalling**	5	HNSC, KICH, LIHC, LUSC, PCPG
**PI3K Cascade**	5	COAD, HNSC, LIHC, LUSC, SARC
**PTK6 Regulates RHO GTPases, RAS GTPase and MAP Kinases**	5	BRCA, COAD, HNSC, KIRP, OV
**Platelet Aggregation (Plug Formation)**	5	DBCL, KIRC, LUAD, LUSC, OV
**RHO GTPase Effectors**	5	BRCA, COAD, LUSC, PCPG, PRAD
**Regulation of CDH11 Function**	5	BRCA, COAD, HNSC, KIRP, PRAD
**Regulation of Localization of FOXO Transcription Factors**	5	COAD, HNSC, LUSC, PCPG, PRAD
**Regulation of NFE2L2 Gene Expression**	5	COAD, HNSC, LUSC, OV, TGCT
**Release of Apoptotic Factors From the Mitochondria**	5	KICH, LIHC, PCPG, PRAD, UCEC
**SARS-CoV-2-host Interactions**	5	COAD, HNSC, KICH, LUSC, PCPG
**SCF(Skp2)-mediated Degradation of P27 P21**	5	LIHC, LUAD, LUSC, PRAD, SARC
**Sema4D Induced Cell Migration and Growth-Cone Collapse**	5	COAD, GBM, HNSC, LUSC, THCA
**Sema4D in Semaphorin Signalling**	5	COAD, GBM, HNSC, LUSC, THCA
**Signalling by ALK**	5	HNSC, LIHC, LUSC, OV, SARC

**Signalling by FGFR1**	5	HNSC, KICH, LIHC, LUSC, PCPG
**Signalling by FGFR3**	5	HNSC, KICH, LIHC, LUSC, PCPG
**Signalling by FGFR4**	5	HNSC, KICH, LIHC, LUSC, PCPG
**Signalling by FLT3 Fusion Proteins**	5	COAD, HNSC, KICH, OV, THYM
**Signalling by Membrane-Tethered Fusions of PDGFRA or PDGFRB**	5	COAD, ESCA, KICH, LIHC, THCA
**Signalling by NOTCH**	5	HNSC, KIRP, LUSC, PCPG, SARC
**Signalling by NTRK1 (TRKA)**	5	HNSC, LIHC, PCPG, PRAD, SARC
**Signalling by NTRK3 (TRKC)**	5	LGG, HNSC, LIHC, PCPG, SARC
**Signalling by PDGFR in Disease**	5	COAD, ESCA, HNSC, LIHC, THCA
**Signalling by SCF-KIT**	5	COAD, HNSC, KICH, LIHC, OV
**Signalling by WNT**	5	BRCA, COAD, HNSC, LUSC, OV
**Synthesis of diphthamide-EEF2**	5	BRCA, COAD, KIRP, MESO, SKCM
**TFAP2 (AP-2) Family Regulates Transcription of Cell Cycle Factors**	5	COAD, HNSC, KICH, LUSC, OV
**Translocation of SLC2A4 (GLUT4) to the Plasma Membrane**	5	LGG, COAD, HNSC, LUSC, PRAD
**VEGFR2 Mediated Cell Proliferation**	5	COAD, ESCA, OV, PCPG, THCA
**Viral Infection Pathways**	5	COAD, KICH, KIRP, LUSC, SARC

Negatively associated Reactome Enriched Pathways		
**Term**	**#Tumours**	**Tumour type**
**Diseases of Signal Transduction by Growth Factor Receptors and Second Messengers**	10	BRCA, COAD, KICH, KIRC, KIRP, LIHC, SARC, SKCM, TGCT, THCA
**SARS-CoV-1 Targets Host Intracellular Signalling and Regulatory Pathways**	10	BRCA, COAD, HNSC, KIRC, KIRP, LIHC, LUAD, LUSC, SKCM, THCA
**Signal Transduction**	10	BRCA, COAD, HNSC, KIRC, LIHC, LUSC, SARC, SKCM, TGCT, THCA
**Transcriptional Regulation by TP53**	10	HNSC, KICH, KIRP, LIHC, LUSC, PRAD, SKCM, STAD, TGCT, THCA
**DNA Repair**	9	ESCA, HNSC, KICH, KIRP, LIHC, PRAD, SKCM, STAD, TGCT
**Defective Intrinsic Pathway for Apoptosis**	9	COAD, KICH, KIRC, KIRP, LIHC, LUAD, LUSC, SKCM, THCA
**FOXO-mediated Transcription of Cell Cycle Genes**	9	BRCA, COAD, HNSC, KICH, KIRC, KIRP, LIHC, SKCM, THCA
**RNA Polymerase II Transcription**	9	COAD, HNSC, KICH, KIRP, LIHC, PRAD, SKCM, TGCT, THCA
**RUNX3 Regulates BCL2L11 (BIM) Transcription**	9	BRCA, COAD, HNSC, KICH, KIRC, KIRP, LIHC, SKCM, THCA
**Chk1 Chk2(Cds1) Mediated Inactivation of Cyclin B Cdk1 Complex**	8	HNSC, LIHC, LUAD, LUSC, PRAD, SKCM, TGCT, THCA
**Deregulated CDK5 Triggers Multiple Neurodegenerative Pathways in Alzheimer's Disease Models**	8	COAD, KIRC, KIRP, LIHC, LUAD, LUSC, SKCM, THCA
**ESR-mediated Signalling**	8	BRCA, COAD, KIRC, KIRP, LIHC, SARC, SKCM, THCA
**Extra-nuclear Oestrogen Signalling**	8	BRCA, COAD, KIRC, KIRP, LIHC, SARC, SKCM, THCA
**G2 M DNA Damage Checkpoint**	8	HNSC, KICH, LIHC, LUAD, LUSC, PRAD, SKCM, TGCT
**Generic Transcription Pathway**	8	COAD, HNSC, KICH, KIRP, LIHC, PRAD, SKCM, TGCT
**Neurodegenerative Diseases**	8	COAD, KIRC, KIRP, LIHC, LUAD, LUSC, SKCM, THCA
**RUNX3 Regulates CDKN1A Transcription**	8	BRCA, COAD, HNSC, KICH, KIRC, KIRP, LIHC, TGCT
**Signalling by NODAL**	8	BRCA, COAD, KICH, KIRC, KIRP, LIHC, SKCM, THCA
**Signalling by Nuclear Receptors**	8	BRCA, COAD, KIRC, KIRP, LIHC, SARC, SKCM, THCA
**p38MAPK Events**	8	LGG, BRCA, COAD, KIRC, KIRP, SARC, SKCM, UCEC
**Activation of BAD and Translocation to Mitochondria**	7	HNSC, KIRC, LIHC, LUAD, LUSC, SKCM, THCA
**Cell Cycle Checkpoints**	7	HNSC, LIHC, LUSC, PRAD, SKCM, TGCT, THCA

**Cellular Responses to Stimuli**	7	COAD, KIRP, LIHC, SARC, SKCM, TGCT, THCA
**DNA Double-Strand Break Repair**	7	HNSC, KICH, KIRP, LIHC, SKCM, STAD, TGCT
**Disease**	7	BRCA, COAD, KIRP, LIHC, SARC, SKCM, TGCT
**FLT3 Signalling**	7	BRCA, COAD, KIRP, LIHC, SARC, SKCM, THCA
**G2 M Checkpoints**	7	HNSC, LIHC, LUAD, LUSC, PRAD, SKCM, TGCT
**Gene Expression (Transcription)**	7	COAD, HNSC, KIRP, LIHC, SKCM, TGCT, THCA
**Homology Directed Repair**	7	HNSC, KICH, KIRP, LIHC, SKCM, STAD, TGCT
**MAPK Family Signalling Cascades**	7	BRCA, COAD, KIRP, LIHC, SARC, SKCM, THCA
**PI3K AKT Signalling in Cancer**	7	COAD, KICH, LIHC, SARC, SKCM, TGCT, THCA
**SARS-CoV-1 Infection**	7	BRCA, HNSC, LIHC, LUAD, LUSC, SKCM, TGCT
**Signalling by ERBB4**	7	BRCA, COAD, KIRC, KIRP, LIHC, SARC, SKCM
**Signalling by FLT3 Fusion Proteins**	7	BRCA, COAD, KIRC, KIRP, SARC, SKCM, TGCT
**Signalling by FLT3 ITD and TKD Mutants**	7	BRCA, COAD, KIRC, KIRP, SARC, SKCM, TGCT
**Signalling by NTRK3 (TRKC)**	7	BRCA, COAD, HNSC, KIRC, KIRP, SARC, SKCM
**Signalling by Receptor Tyrosine Kinases**	7	LGG, COAD, HNSC, SARC, SKCM, TGCT, UCEC
**Signalling to RAS**	7	BRCA, COAD, KIRC, KIRP, SARC, SKCM, UCEC
**Apoptosis**	6	HNSC, LIHC, LUAD, LUSC, TGCT, THCA
**Cell Surface Interactions at the Vascular Wall**	6	BRCA, COAD, KIRC, KIRP, SARC, SKCM
**Cellular Responses to Stress**	6	COAD, LIHC, SARC, SKCM, TGCT, THCA
**Constitutive Signalling by AKT1 E17K in Cancer**	6	COAD, KIRP, LIHC, SKCM, TGCT, THCA
**Constitutive Signalling by EGFRvIII**	6	BRCA, COAD, KIRC, KIRP, SARC, SKCM
**Constitutive Signalling by Ligand-Responsive EGFR Cancer Variants**	6	BRCA, COAD, KIRC, KIRP, SARC, SKCM
**Cytokine Signalling in Immune System**	6	LGG, COAD, LIHC, SARC, TGCT, THCA
**Diseases of Mismatch Repair (MMR)**	6	COAD, ESCA, KIRP, LIHC, PRAD, TGCT
**Downstream Signalling of Activated FGFR3**	6	BRCA, COAD, KIRC, KIRP, SARC, SKCM
**Oestrogen-dependent Nuclear Events Downstream of ESR-membrane Signalling**	6	COAD, KIRC, KIRP, LIHC, SKCM, THCA

**FLT3 Signalling in Disease**	6	BRCA, COAD, KIRP, SARC, SKCM, TGCT
**FOXO-mediated Transcription**	6	BRCA, COAD, HNSC, KIRP, LIHC, THCA
**FOXO-mediated Transcription of Oxidative Stress, Metabolic and Neuronal Genes**	6	BRCA, COAD, KICH, KIRP, LIHC, THCA
**HDR Through MMEJ (alt-NHEJ)**	6	HNSC, KICH, KIRP, SKCM, TGCT, THCA
**Mitochondrial Unfolded Protein Response (UPRmt)**	6	COAD, KICH, KIRP, LIHC, SKCM, THCA
**Programmed Cell Death**	6	HNSC, LIHC, LUAD, LUSC, TGCT, THCA
**Regulation of Localization of FOXO Transcription Factors**	6	COAD, HNSC, KIRP, LIHC, SKCM, THCA
**SARS-CoV-2 Targets Host Intracellular Signalling and Regulatory Pathways**	6	HNSC, LIHC, LUAD, LUSC, SKCM, THCA
**Signalling by BMP**	6	BRCA, COAD, KIRC, KIRP, LIHC, LUSC
**Signalling by EGFR in Cancer**	6	BRCA, COAD, KIRC, KIRP, SARC, SKCM
**Signalling by EGFRvIII in Cancer**	6	BRCA, COAD, KIRC, KIRP, SARC, SKCM
**Signalling by ERBB2**	6	BRCA, COAD, KIRC, KIRP, SARC, SKCM
**Signalling by ERBB2 ECD Mutants**	6	BRCA, COAD, KIRC, KIRP, SARC, SKCM
**Signalling by ERBB2 KD Mutants**	6	BRCA, COAD, KIRC, KIRP, SARC, SKCM
**Signalling by ERBB2 in Cancer**	6	BRCA, COAD, KIRC, KIRP, SARC, SKCM
**Signalling by Erythropoietin**	6	BRCA, COAD, KIRC, KIRP, SARC, SKCM
**Signalling by FGFR3 in Disease**	6	BRCA, COAD, KIRC, KIRP, SARC, SKCM
**Signalling by FGFR4 in Disease**	6	BRCA, COAD, KIRC, KIRP, SARC, SKCM
**Signalling by Hippo**	6	COAD, LIHC, LUAD, LUSC, SKCM, THCA
**Signalling by KIT in Disease**	6	BRCA, COAD, KIRC, KIRP, SARC, SKCM
**Signalling by Ligand-Responsive EGFR Variants in Cancer**	6	BRCA, COAD, KIRC, KIRP, SARC, SKCM
**Signalling by NTRK2 (TRKB)**	6	BRCA, COAD, KIRC, KIRP, SARC, SKCM
**Signalling by NTRKs**	6	LGG, BRCA, COAD, SARC, SKCM, UCEC
**Signalling by PDGFR in Disease**	6	BRCA, COAD, KIRC, KIRP, SARC, SKCM
**Signalling by PDGFRA Extracellular Domain Mutants**	6	BRCA, COAD, KIRC, KIRP, SARC, SKCM

**Signalling by PDGFRA Transmembrane, Juxtamembrane and Kinase Domain Mutants**	6	BRCA, COAD, KIRC, KIRP, SARC, SKCM
**Signalling by Phosphorylated Juxtamembrane, Extracellular and Kinase Domain KIT Mutants**	6	BRCA, COAD, KIRC, KIRP, SARC, SKCM
**Signalling by SCF-KIT**	6	BRCA, COAD, KIRP, SARC, SKCM, TGCT
**Signalling to ERKs**	6	BRCA, COAD, KIRP, SARC, THCA, UCEC
**Tie2 Signalling**	6	BRCA, COAD, KIRC, KIRP, SARC, SKCM
**Transcriptional Regulation by RUNX2**	6	BRCA, COAD, HNSC, LIHC, PRAD, TGCT
**Aberrant Regulation of Mitotic G1 S Transition in Cancer Due to RB1 Defects**	5	COAD, HNSC, LIHC, TGCT, THCA
**Activated NTRK2 Signals Through FRS2 and FRS3**	5	BRCA, COAD, KIRC, KIRP, SKCM
**Activated NTRK2 Signals Through RAS**	5	BRCA, COAD, KIRC, KIRP, SKCM
**Activated NTRK3 Signals Through RAS**	5	BRCA, COAD, KIRC, KIRP, SKCM
**Activation of RAS in B Cells**	5	BRCA, COAD, KIRC, KIRP, SKCM
**CD209 (DC-SIGN) Signalling**	5	BRCA, COAD, KIRC, KIRP, SKCM
**Cell Cycle**	5	HNSC, LIHC, PRAD, SKCM, TGCT
**Cell Death Signalling via NRAGE, NRIF and NADE**	5	ESCA, LUAD, LUSC, SARC, THCA
**Constitutive Signalling by Overexpressed ERBB2**	5	BRCA, COAD, KIRC, KIRP, SKCM
**DAP12 Interactions**	5	BRCA, COAD, KIRP, SARC, SKCM
**DAP12 Signalling**	5	BRCA, COAD, KIRP, SARC, SKCM
**Death Receptor Signalling**	5	ESCA, LUAD, LUSC, SARC, THCA
**Defective Binding of RB1 Mutants to E2F1,(E2F2, E2F3)**	5	COAD, HNSC, LIHC, TGCT, THCA
**Diseases of DNA Repair**	5	ESCA, KIRP, LIHC, PRAD, SKCM
**Downstream Signal Transduction**	5	BRCA, COAD, KIRP, SARC, SKCM
**Downstream Signalling of Activated FGFR1**	5	BRCA, COAD, KIRP, SARC, SKCM
**Downstream Signalling of Activated FGFR2**	5	BRCA, COAD, KIRP, SARC, SKCM
**Downstream Signalling of Activated FGFR4**	5	BRCA, COAD, KIRP, SARC, SKCM
**EGFR Transactivation by Gastrin**	5	BRCA, COAD, KIRC, KIRP, SKCM

**Erythropoietin Activates RAS**	5	BRCA, COAD, KIRC, KIRP, SKCM
**Oestrogen-stimulated Signalling Through PRKCZ**	5	BRCA, COAD, KIRC, KIRP, SKCM
**FRS-mediated FGFR1 Signalling**	5	BRCA, COAD, KIRC, KIRP, SKCM
**FRS-mediated FGFR2 Signalling**	5	BRCA, COAD, KIRC, KIRP, SKCM
**FRS-mediated FGFR3 Signalling**	5	BRCA, COAD, KIRC, KIRP, SKCM
**FRS-mediated FGFR4 Signalling**	5	BRCA, COAD, KIRC, KIRP, SKCM
**Formation of Definitive Endoderm**	5	BRCA, COAD, HNSC, KIRC, KIRP
**GRB2 Events in EGFR Signalling**	5	BRCA, COAD, KIRC, KIRP, SKCM
**GRB2 Events in ERBB2 Signalling**	5	BRCA, COAD, KIRC, KIRP, SKCM
**Gastrin-CREB Signalling Pathway via PKC and MAPK**	5	BRCA, COAD, KIRC, KIRP, SKCM
**Germ Layer Formation at Gastrulation**	5	BRCA, COAD, HNSC, KIRC, KIRP
**HDR Through Homologous Recombination (HRR)**	5	HNSC, KIRP, LIHC, SKCM, STAD
**HDR Through Homologous Recombination (HRR) or Single Strand Annealing (SSA)**	5	HNSC, KIRP, LIHC, SKCM, STAD
**IGF1R Signalling Cascade**	5	BRCA, COAD, KIRP, SARC, SKCM
**IRS-mediated Signalling**	5	BRCA, COAD, KIRP, SARC, SKCM
**IRS-related Events Triggered by IGF1R**	5	BRCA, COAD, KIRP, SARC, SKCM
**Insulin Receptor Signalling Cascade**	5	BRCA, COAD, KIRP, SARC, SKCM
**Intracellular Signalling by Second Messengers**	5	COAD, LIHC, SARC, TGCT, THCA
**Intrinsic Pathway for Apoptosis**	5	HNSC, LIHC, LUAD, LUSC, TGCT
**Loss of Function of SMAD2 3 in Cancer**	5	BRCA, COAD, HNSC, KIRC, KIRP
**MET Activates RAS Signalling**	5	BRCA, COAD, KIRC, KIRP, SKCM
**Mismatch Repair**	5	COAD, ESCA, KIRP, PRAD, STAD
**Mismatch Repair (MMR) Directed by MSH2 MSH3 (MutSbeta)**	5	COAD, ESCA, KIRP, PRAD, STAD
**Mismatch Repair (MMR) Directed by MSH2 MSH6 (MutSalpha)**	5	COAD, ESCA, KIRP, PRAD, STAD
**P75 NTR Receptor-Mediated Signalling**	5	ESCA, LUAD, LUSC, SARC, THCA

**PIP3 Activates AKT Signalling**	5	COAD, LIHC, SARC, TGCT, THCA
**PTK6 Regulates RHO GTPases, RAS GTPase and MAP Kinases**	5	BRCA, COAD, KIRC, KIRP, SKCM
**Presynaptic Phase of Homologous DNA Pairing and Strand Exchange**	5	HNSC, KIRP, LIHC, PRAD, SKCM
**RAS Processing**	5	BRCA, COAD, KIRC, KIRP, SKCM
**RAS Signalling Downstream of NF1 Loss-Of-Function Variants**	5	BRCA, COAD, KIRC, KIRP, SKCM
**Ras Activation Upon Ca2+ Influx Through NMDA Receptor**	5	BRCA, COAD, KIRC, KIRP, SKCM
**Regulation of TP53 Activity**	5	KICH, LIHC, SKCM, TGCT, UCEC
**Regulation of TP53 Activity Through Phosphorylation**	5	KICH, SARC, SKCM, TGCT, UCEC
**SARS-CoV-1-host Interactions**	5	BRCA, HNSC, LIHC, LUAD, LUSC
**SHC-mediated Cascade FGFR1**	5	BRCA, COAD, KIRC, KIRP, SKCM
**SHC-mediated Cascade FGFR2**	5	BRCA, COAD, KIRC, KIRP, SKCM
**SHC-mediated Cascade FGFR3**	5	BRCA, COAD, KIRC, KIRP, SKCM
**SHC-mediated Cascade FGFR4**	5	BRCA, COAD, KIRC, KIRP, SKCM
**SHC-related Events Triggered by IGF1R**	5	BRCA, COAD, KIRC, KIRP, SKCM
**SHC1 Events in EGFR Signalling**	5	BRCA, COAD, KIRC, KIRP, SKCM
**SHC1 Events in ERBB2 Signalling**	5	BRCA, COAD, KIRC, KIRP, SKCM
**SHC1 Events in ERBB4 Signalling**	5	BRCA, COAD, KIRC, KIRP, SKCM
**SOS-mediated Signalling**	5	BRCA, COAD, KIRC, KIRP, SKCM
**Signalling by Activin**	5	BRCA, COAD, HNSC, KIRC, KIRP
**Signalling by EGFR**	5	BRCA, COAD, KIRP, SARC, SKCM
**Signalling by ERBB2 TMD JMD Mutants**	5	BRCA, COAD, KIRC, KIRP, SKCM
**Signalling by FGFR1**	5	BRCA, COAD, KIRP, SARC, SKCM
**Signalling by FGFR1 in Disease**	5	BRCA, COAD, KIRP, SARC, SKCM
**Signalling by FGFR2 in Disease**	5	BRCA, COAD, KIRP, SARC, SKCM
**Signalling by FGFR3**	5	BRCA, COAD, KIRP, SARC, SKCM

**Signalling by FGFR4**	5	BRCA, COAD, KIRP, SARC, SKCM
**Signalling by NTRK1 (TRKA)**	5	BRCA, COAD, SARC, SKCM, UCEC
**Signalling by Non-Receptor Tyrosine Kinases**	5	BRCA, COAD, KIRC, KIRP, LIHC
**Signalling by PDGF**	5	BRCA, COAD, KIRP, SARC, SKCM
**Signalling by PTK6**	5	BRCA, COAD, KIRC, KIRP, LIHC
**Signalling by TGF-beta Receptor Complex in Cancer**	5	BRCA, COAD, HNSC, KIRC, KIRP
**Signalling by Type 1 Insulin-like Growth Factor 1 Receptor (IGF1R)**	5	BRCA, COAD, KIRP, SARC, SKCM
**Signalling by VEGF**	5	BRCA, COAD, SARC, SKCM, UCEC
**TP53 Regulates Metabolic Genes**	5	LIHC, LUAD, LUSC, SKCM, TGCT
**TP53 Regulates Transcription of Cell Cycle Genes**	5	HNSC, LIHC, PRAD, STAD, TGCT
**TP53 Regulates Transcription of Genes Involved in G1 Cell Cycle Arrest**	5	COAD, HNSC, LIHC, TGCT, THCA
**TP53 Regulates Transcription of Genes Involved in G2 Cell Cycle Arrest**	5	HNSC, LIHC, PRAD, STAD, TGCT
**Transcriptional Regulation by RUNX3**	5	BRCA, COAD, KICH, KIRP, LIHC
**VEGFA-VEGFR2 Pathway**	5	BRCA, COAD, SARC, SKCM, UCEC
**VEGFR2 Mediated Cell Proliferation**	5	BRCA, COAD, KIRC, KIRP, SKCM
