# Supplementary material for: YBX1 Expression Marks Proliferative Tumour States with Context-Dependent Genomic Instability: A Pan-Cancer Analysis
Source: Int J Mol Sci. 2026 May 13;27(10):4340. doi: 10.3390/ijms27104340 (PMC13207732; doi:10.3390/ijms27104340)
Supplement: Supplementary file 1 [file ijms-27-04340-s001.zip › Supplementary Table S3_V1_280426.pdf]

Table S3. Biological processes enriched among genes frequently mutated in high-*YBX1* and low-*YBX1* tumours, and those common to both, identified using EnrichR.

**Term (high *YBX1* exclusive)**	**P-value (high *YBX1*)**	**Adjusted P-value (high *YBX1*)**	**P-value (low *YBX1*)**	**Adjusted P-value (low *YBX1*)**
**Positive Regulation of Cell Migration (GO:0030335)**	3.52E-08	9.12E-06	-	-
**Chromatin Remodeling (GO:0006338)**	2.31E-07	4.26E-05	-	-
**Mammary Gland Epithelium Development (GO:0061180)**	4.60E-07	7.44E-05	-	-
**Positive Regulation of Cell Differentiation (GO:0045597)**	7.28E-07	1.05E-04	-	-
**Chromatin Organization (GO:0006325)**	1.56E-06	1.83E-04	-	-
**DNA Damage Response (GO:0006974)**	2.36E-06	2.54E-04	-	-
**Mismatch Repair (GO:0006298)**	3.48E-06	3.09E-04	-	-
**Cellular Response to Mechanical Stimulus (GO:0071260)**	3.57E-06	3.09E-04	-	-
**Negative Regulation of Transcription by RNA Polymerase II (GO:0000122)**	3.58E-06	3.09E-04	-	-
**Positive Regulation of RNA Biosynthetic Process (GO:1902680)**	5.58E-06	4.51E-04	-	-
**Negative Regulation of Programmed Cell Death (GO:0043069)**	6.90E-06	4.70E-04	-	-
**Regulation of Apoptotic Process (GO:0042981)**	1.20E-05	7.42E-04	-	-
**Enzyme-Linked Receptor Protein Signaling Pathway (GO:0007167)**	1.57E-05	9.21E-04	-	-
**Positive Regulation of Gene Expression (GO:0010628)**	2.17E-05	1.22E-03	-	-
**Positive Regulation of Macromolecule Biosynthetic Process (GO:0010557)**	2.63E-05	1.42E-03	-	-
**Regulation of Interleukin-1 Beta Production (GO:0032651)**	3.07E-05	1.53E-03	-	-
**Negative Regulation of Myoblast Differentiation (GO:0045662)**	4.42E-05	2.00E-03	-	-
**Regulation of Nitric Oxide Biosynthetic Process (GO:0045428)**	4.54E-05	2.00E-03	-	-
**Cellular Response to Oxygen-Containing Compound (GO:1901701)**	4.85E-05	2.00E-03	-	-
**Regulation of Fibroblast Proliferation (GO:0048145)**	4.96E-05	2.00E-03	-	-
**Development of Primary Male Sexual Characteristics (GO:0046546)**	4.96E-05	2.00E-03	-	-
**Male Gonad Development (GO:0008584)**	4.96E-05	2.00E-03	-	-
**Chromosome Organization (GO:0051276)**	5.40E-05	2.12E-03	-	-
**Regulation of Autophagy (GO:0010506)**	6.99E-05	2.58E-03	-	-

**Apoptotic Process (GO:0006915)**	7.38E-05	2.58E-03	-	-
**Vasculature Development (GO:0001944)**	7.58E-05	2.58E-03	-	-
**Maintenance of Gastrointestinal Epithelium (GO:0030277)**	7.58E-05	2.58E-03	-	-
**Mitotic Sister Chromatid Cohesion (GO:0007064)**	7.58E-05	2.58E-03	-	-
**Regulation of Cell Cycle (GO:0051726)**	7.98E-05	2.65E-03	-	-
**Positive Regulation of miRNA Metabolic Process (GO:2000630)**	8.69E-05	2.78E-03	-	-
**Positive Regulation of Cellular Process (GO:0048522)**	8.80E-05	2.78E-03	-	-
**Gonad Development (GO:0008406)**	9.35E-05	2.88E-03	-	-
**Regulation of Cytokine Production Involved in Inflammatory Response (GO:1900015)**	1.01E-04	2.99E-03	-	-
**Cellular Response to Nitrogen Compound (GO:1901699)**	1.02E-04	2.99E-03	-	-
**Positive Regulation of Interleukin-1 Beta Production (GO:0032731)**	1.08E-04	3.10E-03	-	-
**Negative Regulation of Cellular Process (GO:0048523)**	1.13E-04	3.14E-03	-	-
**Heart Morphogenesis (GO:0003007)**	1.16E-04	3.14E-03	-	-
**Positive Regulation of Interferon-Alpha Production (GO:0032727)**	1.19E-04	3.14E-03	-	-
**Mammary Gland Development (GO:0030879)**	1.19E-04	3.14E-03	-	-
**Regulation of Myoblast Differentiation (GO:0045661)**	1.24E-04	3.14E-03	-	-
**Peptidyl-Tyrosine Phosphorylation (GO:0018108)**	1.24E-04	3.14E-03	-	-
**Regulation of G1/S Transition of Mitotic Cell Cycle (GO:2000045)**	1.31E-04	3.25E-03	-	-
**Regulation of Interleukin-6 Production (GO:0032675)**	1.48E-04	3.57E-03	-	-
**Negative Regulation of Growth (GO:0045926)**	1.54E-04	3.57E-03	-	-
**Regulation of Lipid Storage (GO:0010883)**	1.56E-04	3.57E-03	-	-
**Positive Regulation of NLRP3 Inflammasome Complex Assembly (GO:1900227)**	1.56E-04	3.57E-03	-	-
**Positive Regulation of Catabolic Process (GO:0009896)**	1.57E-04	3.57E-03	-	-
**Positive Regulation of Interleukin-1 Production (GO:0032732)**	1.71E-04	3.78E-03	-	-
**Positive Regulation of Inflammasome-Mediated Signaling Pathway (GO:0141087)**	1.76E-04	3.78E-03	-	-
**G1/S Transition of Mitotic Cell Cycle (GO:0000082)**	1.82E-04	3.78E-03	-	-

**Regulation of Pentose-Phosphate Shunt (GO:0043456)**	1.90E-04	3.78E-03	-	-
**Reproductive System Development (GO:0061458)**	1.90E-04	3.78E-03	-	-
**Positive Regulation of Gonad Development (GO:1905941)**	1.90E-04	3.78E-03	-	-
**Positive Regulation of Male Gonad Development (GO:2000020)**	1.90E-04	3.78E-03	-	-
**Maintenance of DNA Repeat Elements (GO:0043570)**	1.90E-04	3.78E-03	-	-
**Negative Regulation of Cell Growth (GO:0030308)**	1.96E-04	3.84E-03	-	-
**Regulation of miRNA Transcription (GO:1902893)**	2.05E-04	3.87E-03	-	-
**Cell Cycle G1/S Phase Transition (GO:0044843)**	2.05E-04	3.87E-03	-	-
**Regulation of RNA Biosynthetic Process (GO:2001141)**	2.07E-04	3.87E-03	-	-
**Response to Cytokine (GO:0034097)**	2.11E-04	3.91E-03	-	-
**Regulation of Interferon-Alpha Production (GO:0032647)**	2.23E-04	4.06E-03	-	-
**Ras Protein Signal Transduction (GO:0007265)**	2.30E-04	4.07E-03	-	-
**Positive Regulation of Type I Interferon Production (GO:0032481)**	2.30E-04	4.07E-03	-	-
**Positive Regulation of Fibroblast Proliferation (GO:0048146)**	2.49E-04	4.35E-03	-	-
**Regulation of Translation (GO:0006417)**	2.61E-04	4.51E-03	-	-
**Regulation of Platelet Activation (GO:0010543)**	2.77E-04	4.59E-03	-	-
**Regulation of Tumor Necrosis Factor Production (GO:0032680)**	2.83E-04	4.59E-03	-	-
**Regulation of Male Gonad Development (GO:2000018)**	2.84E-04	4.59E-03	-	-
**Regulation of Osteoclast Development (GO:2001204)**	2.84E-04	4.59E-03	-	-
**Establishment of Mitotic Sister Chromatid Cohesion (GO:0034087)**	2.84E-04	4.59E-03	-	-
**Circulatory System Development (GO:0072359)**	2.93E-04	4.68E-03	-	-
**Gland Development (GO:0048732)**	3.35E-04	5.29E-03	-	-
**Regulation of Cytoskeleton Organization (GO:0051493)**	3.72E-04	5.75E-03	-	-
**DNA Repair (GO:0006281)**	3.73E-04	5.75E-03	-	-
**Mammary Gland Epithelial Cell Differentiation (GO:0060644)**	3.96E-04	6.03E-03	-	-
**Negative Regulation of Cell Differentiation (GO:0045596)**	4.23E-04	6.37E-03	-	-
**Regulation of Cell Growth (GO:0001558)**	4.44E-04	6.56E-03	-	-
**Regulation of Cell Cycle Phase Transition (GO:1901987)**	4.46E-04	6.56E-03	-	-
**Regulation of ERK1 and ERK2 Cascade (GO:0070372)**	4.55E-04	6.61E-03	-	-

**Positive Regulation of Tumor Necrosis Factor Production (GO:0032760)**	4.71E-04	6.77E-03	-	-
**Regulation of Interleukin-17 Production (GO:0032660)**	4.87E-04	6.84E-03	-	-
**Platelet Aggregation (GO:0070527)**	4.87E-04	6.84E-03	-	-
**Regulation of Interleukin-23 Production (GO:0032667)**	5.27E-04	7.03E-03	-	-
**Anoikis (GO:0043276)**	5.27E-04	7.03E-03	-	-
**Somatic Recombination of Immunoglobulin Gene Segments (GO:0016447)**	5.27E-04	7.03E-03	-	-
**Embryonic Digestive Tract Morphogenesis (GO:0048557)**	5.27E-04	7.03E-03	-	-
**Cellular Response to Growth Factor Stimulus (GO:0071363)**	5.27E-04	7.03E-03	-	-
**Positive Regulation of Tumor Necrosis Factor Superfamily Cytokine Production (GO:1903557)**	5.40E-04	7.13E-03	-	-
**Regulation of Endothelial Cell Migration (GO:0010594)**	6.16E-04	7.97E-03	-	-
**Regulation of Mitotic Cell Cycle Phase Transition (GO:1901990)**	6.16E-04	7.97E-03	-	-
**Embryonic Forelimb Morphogenesis (GO:0035115)**	6.75E-04	8.65E-03	-	-
**Regulation of Cytokine-Mediated Signaling Pathway (GO:0001959)**	7.23E-04	9.08E-03	-	-
**Regulation of DNA Recombination (GO:0000018)**	7.23E-04	9.08E-03	-	-
**Regulation of Inflammatory Response (GO:0050727)**	8.11E-04	9.96E-03	-	-
**Regulation of NLRP3 Inflammasome Complex Assembly (GO:1900225)**	8.35E-04	9.96E-03	-	-
**Regulation of Mesenchymal Stem Cell Differentiation (GO:2000739)**	8.42E-04	9.96E-03	-	-
**Cellular Response to Oxidised Low-Density Lipoprotein Particle Stimulus (GO:0140052)**	8.42E-04	9.96E-03	-	-
**Response to X-ray (GO:0010165)**	8.42E-04	9.96E-03	-	-
**Forelimb Morphogenesis (GO:0035136)**	8.42E-04	9.96E-03	-	-
**B Cell Activation (GO:0042113)**	8.54E-04	9.96E-03	-	-
**Regulation of Actin Filament-Based Process (GO:0032970)**	8.54E-04	9.96E-03	-	-
**Positive Regulation of Signal Transduction (GO:0009967)**	9.36E-04	1.08E-02	-	-
**Heart Development (GO:0007507)**	9.53E-04	1.08E-02	-	-
**Regulation of Microtubule-Based Process (GO:0032886)**	9.57E-04	1.08E-02	-	-
**Positive Regulation of Autophagy (GO:0010508)**	9.58E-04	1.08E-02	-	-
**Response to UV (GO:0009411)**	9.95E-04	1.11E-02	-	-

**Positive Regulation of Execution Phase of Apoptosis (GO:1900119)**	1.03E-03	1.12E-02	-	-
**Positive Regulation of Platelet Activation (GO:0010572)**	1.03E-03	1.12E-02	-	-
**Positive Regulation of Platelet Aggregation (GO:1901731)**	1.03E-03	1.12E-02	-	-
**Positive Regulation of MAPK Cascade (GO:0043410)**	1.09E-03	1.15E-02	-	-
**Peptidyl-Tyrosine Modification (GO:0018212)**	1.09E-03	1.15E-02	-	-
**Homotypic Cell-Cell Adhesion (GO:0034109)**	1.09E-03	1.15E-02	-	-
**Regulation of Defense Response (GO:0031347)**	1.11E-03	1.16E-02	-	-
**Negative Regulation of Gene Expression (GO:0010629)**	1.12E-03	1.16E-02	-	-
**Regulation of Actin Cytoskeleton Organization (GO:0032956)**	1.15E-03	1.16E-02	-	-
**Negative Regulation of Cold-Induced Thermogenesis (GO:0120163)**	1.16E-03	1.16E-02	-	-
**Stem Cell Differentiation (GO:0048863)**	1.16E-03	1.16E-02	-	-
**Positive Regulation of miRNA Transcription (GO:1902895)**	1.16E-03	1.16E-02	-	-
**Positive Regulation of Protein-Containing Complex Assembly (GO:0031334)**	1.19E-03	1.19E-02	-	-
**Axonal Fasciculation (GO:0007413)**	1.23E-03	1.20E-02	-	-
**Interleukin-6-Mediated Signaling Pathway (GO:0070102)**	1.23E-03	1.20E-02	-	-
**Regulation of Canonical NF-kappaB Signal Transduction (GO:0043122)**	1.23E-03	1.20E-02	-	-
**Protein Phosphorylation (GO:0006468)**	1.29E-03	1.25E-02	-	-
**Positive Regulation of Cell Population Proliferation (GO:0008284)**	1.31E-03	1.26E-02	-	-
**Positive Regulation of Translation (GO:0045727)**	1.32E-03	1.26E-02	-	-
**Small GTPase-mediated Signal Transduction (GO:0007264)**	1.45E-03	1.36E-02	-	-
**Positive Regulation of T Cell Activation (GO:0050870)**	1.46E-03	1.36E-02	-	-
**Regulation of Ubiquitin-Dependent Protein Catabolic Process (GO:2000058)**	1.47E-03	1.36E-02	-	-
**Epithelial to Mesenchymal Transition (GO:0001837)**	1.47E-03	1.36E-02	-	-
**Negative Regulation of Cytokine Production (GO:0001818)**	1.56E-03	1.41E-02	-	-
**Signal Transduction by P53 Class Mediator (GO:0072331)**	1.56E-03	1.41E-02	-	-
**Positive Regulation of Reactive Oxygen Species Metabolic Process (GO:2000379)**	1.56E-03	1.41E-02	-	-
**Mitotic Cell Cycle Phase Transition (GO:0044772)**	1.56E-03	1.41E-02	-	-
**B Cell Differentiation (GO:0030183)**	1.64E-03	1.47E-02	-	-

**Intrinsic Apoptotic Signaling Pathway (GO:0097193)**	1.67E-03	1.47E-02	-	-
**Embryonic Cranial Skeleton Morphogenesis (GO:0048701)**	1.68E-03	1.47E-02	-	-
**Regulation of RNA Export From Nucleus (GO:0046831)**	1.68E-03	1.47E-02	-	-
**Cellular Response to Cytokine Stimulus (GO:0071345)**	1.74E-03	1.51E-02	-	-
**Negative Regulation of Canonical Wnt Signaling Pathway (GO:0090090)**	1.78E-03	1.53E-02	-	-
**Regulation of Execution Phase of Apoptosis (GO:1900117)**	1.94E-03	1.62E-02	-	-
**Negative Regulation of Macrophage Activation (GO:0043031)**	1.94E-03	1.62E-02	-	-
**Positive Regulation of Cytoplasmic Translation (GO:2000767)**	1.94E-03	1.62E-02	-	-
**Genitalia Development (GO:0048806)**	1.94E-03	1.62E-02	-	-
**Mitotic Recombination (GO:0006312)**	1.94E-03	1.62E-02	-	-
**Cell Surface Receptor Protein Tyrosine Kinase Signaling Pathway (GO:0007169)**	1.96E-03	1.63E-02	-	-
**Negative Regulation of Macromolecule Biosynthetic Process (GO:0010558)**	1.99E-03	1.64E-02	-	-
**Cellular Response to Oxidative Stress (GO:0034599)**	2.01E-03	1.64E-02	-	-
**Cellular Response to Interleukin-1 (GO:0071347)**	2.03E-03	1.64E-02	-	-
**mRNA Stabilization (GO:0048255)**	2.03E-03	1.64E-02	-	-
**Eye Development (GO:0001654)**	2.13E-03	1.71E-02	-	-
**Response to UV-B (GO:0010224)**	2.21E-03	1.74E-02	-	-
**Digestive Tract Morphogenesis (GO:0048546)**	2.21E-03	1.74E-02	-	-
**Lipopolysaccharide-Mediated Signaling Pathway (GO:0031663)**	2.21E-03	1.74E-02	-	-
**Response to Interleukin-1 (GO:0070555)**	2.24E-03	1.76E-02	-	-
**Cellular Response to Lipopolysaccharide (GO:0071222)**	2.26E-03	1.76E-02	-	-
**Negative Regulation of MAPK Cascade (GO:0043409)**	2.40E-03	1.86E-02	-	-
**Negative Regulation of Canonical NF-kappaB Signal Transduction (GO:0043124)**	2.46E-03	1.90E-02	-	-
**Regulation of Canonical Wnt Signaling Pathway (GO:0060828)**	2.66E-03	2.04E-02	-	-
**Negative Regulation of Metabolic Process (GO:0009892)**	2.70E-03	2.06E-02	-	-
**Neuron Projection Guidance (GO:0097485)**	2.76E-03	2.07E-02	-	-
**Regulation of Response to External Stimulus (GO:0032101)**	2.76E-03	2.07E-02	-	-

**Positive Regulation of Cell Activation (GO:0050867)**	2.80E-03	2.07E-02	-	-
**Positive Regulation of Cytokine Production Involved in Inflammatory Response (GO:1900017)**	2.80E-03	2.07E-02	-	-
**Positive Regulation of Homotypic Cell-Cell Adhesion (GO:0034112)**	2.80E-03	2.07E-02	-	-
**Regulation of Phosphatidylinositol 3-Kinase/Protein Kinase B Signal Transduction (GO:0051896)**	2.82E-03	2.07E-02	-	-
**Positive Regulation of Mitotic Cell Cycle Phase Transition (GO:1901992)**	2.83E-03	2.07E-02	-	-
**Regulation of Endocytosis (GO:0030100)**	2.95E-03	2.11E-02	-	-
**Regulation of Extrinsic Apoptotic Signaling Pathway (GO:2001236)**	2.95E-03	2.11E-02	-	-
**Positive Regulation of T Cell Proliferation (GO:0042102)**	2.95E-03	2.11E-02	-	-
**Regulation of Cell-Matrix Adhesion (GO:0001952)**	2.95E-03	2.11E-02	-	-
**Embryonic Digestive Tract Development (GO:0048566)**	3.12E-03	2.17E-02	-	-
**Limb Morphogenesis (GO:0035108)**	3.12E-03	2.17E-02	-	-
**Mesoderm Development (GO:0007498)**	3.12E-03	2.17E-02	-	-
**Regulation of Behavior (GO:0050795)**	3.12E-03	2.17E-02	-	-
**Positive Regulation of Cytokine Production (GO:0001819)**	3.12E-03	2.17E-02	-	-
**Negative Regulation of Cytokine-Mediated Signaling Pathway (GO:0001960)**	3.22E-03	2.23E-02	-	-
**Regulation of Necroptotic Process (GO:0060544)**	3.45E-03	2.34E-02	-	-
**Regulation of Nuclear-Transcribed mRNA Catabolic Process, Deadenylation-Dependent Decay (GO:1900151)**	3.45E-03	2.34E-02	-	-
**Positive Regulation of G2/M Transition of Mitotic Cell Cycle (GO:0010971)**	3.45E-03	2.34E-02	-	-
**Regulation of Cell Cycle G2/M Phase Transition (GO:1902749)**	3.45E-03	2.34E-02	-	-
**Positive Regulation of Apoptotic Signaling Pathway (GO:2001235)**	3.64E-03	2.45E-02	-	-
**Cellular Response to Lipid (GO:0071396)**	3.74E-03	2.50E-02	-	-
**Phagocytosis (GO:0006909)**	3.79E-03	2.50E-02	-	-
**Response to Ionizing Radiation (GO:0010212)**	3.79E-03	2.50E-02	-	-
**Regulation of Transcription Regulatory Region DNA Binding (GO:2000677)**	3.81E-03	2.50E-02	-	-
**Regulation of Cell-Substrate Junction Assembly (GO:0090109)**	3.81E-03	2.50E-02	-	-
**Negative Regulation of Wnt Signaling Pathway (GO:0030178)**	3.95E-03	2.58E-02	-	-

**Regulation of Cold-Induced Thermogenesis (GO:0120161)**	4.15E-03	2.70E-02	-	-
**Fc-epsilon Receptor Signaling Pathway (GO:0038095)**	4.18E-03	2.70E-02	-	-
**Negative Regulation of Apoptotic Signaling Pathway (GO:2001234)**	4.26E-03	2.73E-02	-	-
**Negative Regulation of Multicellular Organismal Process (GO:0051241)**	4.39E-03	2.80E-02	-	-
**Negative Regulation of Osteoclast Differentiation (GO:0045671)**	4.56E-03	2.82E-02	-	-
**Cellular Response to Interleukin-6 (GO:0071354)**	4.56E-03	2.82E-02	-	-
**Positive Regulation of Cell Cycle G2/M Phase Transition (GO:1902751)**	4.56E-03	2.82E-02	-	-
**Eye Morphogenesis (GO:0048592)**	4.56E-03	2.82E-02	-	-
**Modulation by Host of Viral Genome Replication (GO:0044827)**	4.56E-03	2.82E-02	-	-
**Axon Guidance (GO:0007411)**	4.56E-03	2.82E-02	-	-
**Lymphocyte Differentiation (GO:0030098)**	4.59E-03	2.83E-02	-	-
**Positive Regulation of ERK1 and ERK2 Cascade (GO:0070374)**	4.67E-03	2.85E-02	-	-
**Regulation of Non-Canonical NF-kappaB Signal Transduction (GO:1901222)**	4.76E-03	2.85E-02	-	-
**Nucleosome Organization (GO:0034728)**	4.76E-03	2.85E-02	-	-
**Cellular Response to UV (GO:0034644)**	4.76E-03	2.85E-02	-	-
**Positive Regulation of Interleukin-6 Production (GO:0032755)**	4.76E-03	2.85E-02	-	-
**Positive Regulation of Lymphocyte Proliferation (GO:0050671)**	4.76E-03	2.85E-02	-	-
**Positive Regulation of Cell Motility (GO:2000147)**	4.87E-03	2.90E-02	-	-
**Cell Chemotaxis (GO:0060326)**	4.94E-03	2.92E-02	-	-
**Adaptive Imm Resp Based on Som Recomb of Imm Rcptrs Built Frm IgSF Domains (GO:0002460)**	4.96E-03	2.92E-02	-	-
**Metanephros Development (GO:0001656)**	4.96E-03	2.92E-02	-	-
**Regulation of Cell Cycle G1/S Phase Transition (GO:1902806)**	5.11E-03	2.99E-02	-	-
**Response to Tumor Necrosis Factor (GO:0034612)**	5.30E-03	3.08E-02	-	-
**Cytokine-Mediated Signaling Pathway (GO:0019221)**	5.38E-03	3.08E-02	-	-
**Negative Regulation of Non-Canonical NF-kappaB Signal Transduction (GO:1901223)**	5.38E-03	3.08E-02	-	-
**Cellular Response to Low-Density Lipoprotein Particle Stimulus (GO:0071404)**	5.38E-03	3.08E-02	-	-
**Positive Regulation of Interleukin-17 Production (GO:0032740)**	5.38E-03	3.08E-02	-	-

**Wound Healing (GO:0042060)**	5.48E-03	3.13E-02	-	-
**+ Reg of Phosphatidylinositol 3-Kinase/Prot Kinase B Signal Transduction (GO:0051897)**	5.71E-03	3.20E-02	-	-
**Regulation of Cytoplasmic Translation (GO:2000765)**	5.81E-03	3.20E-02	-	-
**Negative Regulation of Protein Localization to Nucleus (GO:1900181)**	5.81E-03	3.20E-02	-	-
**Regulation of Platelet Aggregation (GO:0090330)**	5.81E-03	3.20E-02	-	-
**Blood Vessel Endothelial Cell Migration (GO:0043534)**	5.81E-03	3.20E-02	-	-
**Regulation of Actin Polymerization or Depolymerization (GO:0008064)**	5.81E-03	3.20E-02	-	-
**Regulation of Cell Size (GO:0008361)**	5.81E-03	3.20E-02	-	-
**Regulation of Interleukin-8 Production (GO:0032677)**	5.87E-03	3.20E-02	-	-
**Negative Regulation of Protein Phosphorylation (GO:0001933)**	5.87E-03	3.20E-02	-	-
**Regulation of T Cell Proliferation (GO:0042129)**	5.87E-03	3.20E-02	-	-
**Wound Healing, Spreading of Cells (GO:0044319)**	6.25E-03	3.40E-02	-	-
**Cellular Response to Tumor Necrosis Factor (GO:0071356)**	6.69E-03	3.55E-02	-	-
**Positive Regulation of Biosynthetic Process (GO:0009891)**	6.69E-03	3.55E-02	-	-
**Negative Regulation of Type II Interferon Production (GO:0032689)**	6.72E-03	3.55E-02	-	-
**Toll-Like Receptor 4 Signaling Pathway (GO:0034142)**	6.72E-03	3.55E-02	-	-
**Hematopoietic Progenitor Cell Differentiation (GO:0002244)**	6.72E-03	3.55E-02	-	-
**Brain Development (GO:0007420)**	6.75E-03	3.55E-02	-	-
**Regulation of Type II Interferon Production (GO:0032649)**	6.90E-03	3.60E-02	-	-
**Positive Regulation of Endothelial Cell Migration (GO:0010595)**	6.90E-03	3.60E-02	-	-
**DNA-templated Transcription Elongation (GO:0006354)**	7.19E-03	3.71E-02	-	-
**Transcription Elongation by RNA Polymerase II (GO:0006368)**	7.19E-03	3.71E-02	-	-
**mRNA Splice Site Recognition (GO:0006376)**	7.19E-03	3.71E-02	-	-
**Regulation of Epithelial to Mesenchymal Transition (GO:0010717)**	7.57E-03	3.83E-02	-	-
**Negative Regulation of Signal Transduction (GO:0009968)**	7.57E-03	3.83E-02	-	-
**Negative Regulation of Cytokine Production Involved in Inflammatory Response (GO:1900016)**	7.69E-03	3.83E-02	-	-
**Fc Receptor Signaling Pathway (GO:0038093)**	7.69E-03	3.83E-02	-	-

**Cell Surface Toll-Like Receptor Signaling Pathway (GO:0140895)**	7.69E-03	3.83E-02	-	-
**Positive Regulation of Calcium-Mediated Signaling (GO:0050850)**	7.69E-03	3.83E-02	-	-
**Positive Regulation of Catalytic Activity (GO:0043085)**	7.69E-03	3.83E-02	-	-
**Positive Regulation of Nitric Oxide Biosynthetic Process (GO:0045429)**	7.69E-03	3.83E-02	-	-
**Pyroptotic Inflammatory Response (GO:0070269)**	7.69E-03	3.83E-02	-	-
**Regulation of Osteoblast Differentiation (GO:0045667)**	7.80E-03	3.87E-02	-	-
**Regulation of Dendritic Spine Morphogenesis (GO:0061001)**	8.19E-03	3.99E-02	-	-
**Regulation of Macrophage Activation (GO:0043030)**	8.19E-03	3.99E-02	-	-
**Positive Regulation of ATP-dependent Activity (GO:0032781)**	8.19E-03	3.99E-02	-	-
**Positive Regulation of Nitric Oxide Metabolic Process (GO:1904407)**	8.19E-03	3.99E-02	-	-
**Positive Regulation of Immune Response (GO:0050778)**	8.51E-03	4.13E-02	-	-
**DNA Damage Response, Signal Transduction by P53 Class Mediator (GO:0030330)**	8.71E-03	4.19E-02	-	-
**Negative Regulation of Interleukin-6 Production (GO:0032715)**	8.71E-03	4.19E-02	-	-
**Central Nervous System Development (GO:0007417)**	8.87E-03	4.25E-02	-	-
**B Cell Receptor Signaling Pathway (GO:0050853)**	9.25E-03	4.42E-02	-	-
**Positive Regulation of Neuron Projection Development (GO:0010976)**	9.53E-03	4.53E-02	-	-
**Cellular Response to Chemical Stress (GO:0062197)**	9.79E-03	4.58E-02	-	-
**Regulation of Developmental Growth (GO:0048638)**	9.80E-03	4.58E-02	-	-
**Regulation of Protein Polymerization (GO:0032271)**	9.80E-03	4.58E-02	-	-
**Tumor Necrosis Factor-Mediated Signaling Pathway (GO:0033209)**	9.80E-03	4.58E-02	-	-
**Regulation of Ras Protein Signal Transduction (GO:0046578)**	9.80E-03	4.58E-02	-	-
**Negative Regulation of Intracellular Signal Transduction (GO:1902532)**	1.00E-02	4.67E-02	-	-
**Hemopoiesis (GO:0030097)**	1.01E-02	4.67E-02	-	-
**Regulation of p38MAPK Cascade (GO:1900744)**	1.04E-02	4.72E-02	-	-
**Response to Transforming Growth Factor Beta (GO:0071559)**	1.04E-02	4.72E-02	-	-
**Toll-Like Receptor Signaling Pathway (GO:0002224)**	1.04E-02	4.72E-02	-	-
**Macrophage Activation (GO:0042116)**	1.04E-02	4.72E-02	-	-
**Regulation of T Cell Mediated Cytotoxicity (GO:0001914)**	1.04E-02	4.72E-02	-	-

**Regulation of Intracellular Signal Transduction (GO:1902531)**	1.07E-02	4.88E-02	-	-
**Sister Chromatid Segregation (GO:0000819)**	1.09E-02	4.92E-02	-	
**Embryonic Limb Morphogenesis (GO:0030326)**	1.09E-02	4.92E-02	-	-
**Positive Regulation of Epithelial Cell Migration (GO:0010634)**	1.09E-02	4.92E-02	-	-

**Term (low *YBX1* exclusive)**	**P-value (high *YBX1*)**	**Adjusted P-value (high *YBX1*)**	**P-value (low *YBX1*)**	**Adjusted P-value (low *YBX1*)**
**Intracellular Signaling Cassette (GO:0141124)**	-	-	7.99E-06	1.63E-03
**Phosphatidylinositol 3-Kinase/Protein Kinase B Signal Transduction (GO:0043491)**	-	-	2.17E-05	1.63E-03
**MAPK Cascade (GO:0000165)**	-	-	2.18E-05	1.63E-03
**Regulation of CD8-positive, Alpha-Beta T Cell Differentiation (GO:0043376)**	-	-	3.95E-05	2.19E-03
**Negative Regulation of Alpha-Beta T Cell Differentiation (GO:0046639)**	-	-	5.26E-05	2.44E-03
**Response to Peptide Hormone (GO:0043434)**	-	-	6.48E-05	2.73E-03
**Regulation of DNA-templated Transcription Initiation (GO:2000142)**	-	-	8.45E-05	3.01E-03
**Regulation of CD4-positive, Alpha-Beta T Cell Differentiation (GO:0043370)**	-	-	1.24E-04	4.09E-03
**Regulation of Peroxisome Proliferator Activated Receptor Signaling Pathway (GO:0035358)**	-	-	1.46E-04	4.15E-03
**Positive Regulation of Alpha-Beta T Cell Differentiation (GO:0046638)**	-	-	1.46E-04	4.15E-03
**Regulation of Fat Cell Differentiation (GO:0045598)**	-	-	1.55E-04	4.15E-03
**Positive Regulation of Protein Localization to Membrane (GO:1905477)**	-	-	1.61E-04	4.15E-03
**Response to Insulin (GO:0032868)**	-	-	2.02E-04	4.92E-03
**Regulation of Epithelial Cell Proliferation (GO:0050678)**	-	-	2.93E-04	6.78E-03
**Positive Regulation of Protein Targeting to Membrane (GO:0090314)**	-	-	3.54E-04	7.79E-03
**Regulation of Protein Targeting to Membrane (GO:0090313)**	-	-	5.56E-04	1.11E-02
**Regulation of Transcription by RNA Polymerase I (GO:0006356)**	-	-	8.57E-04	1.47E-02
**Negative Regulation of Fat Cell Differentiation (GO:0045599)**	-	-	9.13E-04	1.51E-02
**Positive Regulation of Establishment of Protein Localization (GO:1904951)**	-	-	1.03E-03	1.65E-02
**Semaphorin-Plexin Signaling Pathway (GO:0071526)**	-	-	1.36E-03	2.09E-02
**Positive Regulation of Myeloid Leukocyte Differentiation (GO:0002763)**	-	-	1.65E-03	2.45E-02

**Mitotic Spindle Assembly (GO:0090307)**	-	-	1.97E-03	2.68E-02
**Epidermal Growth Factor Receptor Signaling Pathway (GO:0007173)**	-	-	2.13E-03	2.82E-02
**Positive Regulation of Fat Cell Differentiation (GO:0045600)**	-	-	2.22E-03	2.86E-02
**Regulation of Cell Differentiation (GO:0045595)**	-	-	2.72E-03	3.41E-02
**Myeloid Cell Differentiation (GO:0030099)**	-	-	2.88E-03	3.50E-02
**ERBB Signaling Pathway (GO:0038127)**	-	-	2.98E-03	3.53E-02
**Epigenetic Regulation of Gene Expression (GO:0040029)**	-	-	3.61E-03	4.18E-02
**Positive Regulation of MAP Kinase Activity (GO:0043406)**	-	-	3.84E-03	4.33E-02
**Regulation of MAP Kinase Activity (GO:0043405)**	-	-	4.67E-03	4.81E-02
**Spindle Assembly (GO:0051225)**	-	-	4.67E-03	4.81E-02
**Positive Regulation of Protein Serine/Threonine Kinase Activity (GO:0071902)**	-	-	4.92E-03	4.85E-02
**Term (high and low *YBX1* common)**	**P-value (high *YBX1*)**	**Adjusted P-value (high *YBX1*)**	**P-value (low *YBX1*)**	**Adjusted P-value (low *YBX1*)**
**Positive Regulation of DNA-templated Transcription (GO:0045893)**	5.99E-14	7.76E-11	4.83E-06	1.63E-03
**Positive Regulation of Transcription by RNA Polymerase II (GO:0045944)**	3.48E-12	2.10E-09	4.27E-05	2.19E-03
**Regulation of Transcription by RNA Polymerase II (GO:0006357)**	4.87E-12	2.10E-09	2.15E-05	1.63E-03
**Regulation of Gene Expression (GO:0010468)**	2.30E-09	7.45E-07	1.45E-05	1.63E-03
**Negative Regulation of Apoptotic Process (GO:0043066)**	1.54E-07	3.32E-05	4.09E-03	4.41E-02
**Negative Regulation of DNA-templated Transcription (GO:0045892)**	1.54E-06	1.83E-04	3.72E-04	7.84E-03
**Regulation of Cell Population Proliferation (GO:0042127)**	6.30E-06	4.63E-04	5.78E-04	1.11E-02
**Negative Regulation of Cell Population Proliferation (GO:0008285)**	6.44E-06	4.63E-04	1.81E-03	2.54E-02
**Regulation of DNA-templated Transcription (GO:0006355)**	1.15E-05	7.42E-04	7.76E-05	2.99E-03
**Positive Regulation of Intracellular Signal Transduction (GO:1902533)**	2.78E-05	1.44E-03	2.47E-05	1.63E-03
**Negative Regulation of RNA Biosynthetic Process (GO:1902679)**	1.09E-03	1.15E-02	3.94E-03	4.35E-02
**Myeloid Leukocyte Differentiation (GO:0002573)**	4.26E-03	2.73E-02	4.80E-03	4.83E-02
**Cellular Response to Epidermal Growth Factor Stimulus (GO:0071364)**	6.72E-03	3.55E-02	6.99E-04	1.29E-02
**Regulation of Macromolecule Biosynthetic Process (GO:0010556)**	6.75E-03	3.55E-02	1.69E-03	2.45E-02
**Response to Epidermal Growth Factor (GO:0070849)**	8.19E-03	3.99E-02	8.57E-04	1.47E-02
